# A *Listeria ivanovii* balanced‐lethal system may be a promising antigen carrier for vaccine construction

**DOI:** 10.1111/1751-7915.14137

**Published:** 2022-09-07

**Authors:** Yao Lei, Yuzhen Zhou, Yunwen Zhang, Sijing Liu, Sicheng Tian, Qian Ou, Ting Liu, Huan Huang, Tian Tang, Chuan Wang

**Affiliations:** ^1^ West China School of Public Health and West China Fourth Hospital Sichuan University Chengdu China; ^2^ Research Center for Public Health and Preventive Medicine West China School of Public Health, Sichuan University Chengdu China

## Abstract

Expressing heterologous antigens by plasmids may cause antibiotic resistance. Additionally, antigen expression via plasmids is unstable due to the loss of the plasmid. Here, we developed a balanced‐lethal system. The *Listeria monocytogenes* (LM) balanced‐lethal system has been previously used as an antigen carrier to induce cellular immune response. However, thus far, there has been no reports on *Listeria ivanovii* (LI) balanced‐lethal systems. The *dal* and *dat* genes from the LI‐attenuated LIΔ*atcAplcB* (LIΔ) were deleted consecutively, resulting in a nutrient‐deficient LIΔdd strain. Subsequently, an antibiotic resistance‐free plasmid carrying the LM *dal* gene was transformed into the nutrient‐deficient strain to generate the LI balanced‐lethal system LIΔdd:*dal*. The resultant bacterial strain retains the ability to proliferate in phagocytic cells, as well as the ability to adhere and invade hepatocytes. Its genetic composition was stable, and compared to the parent strain, the balanced‐lethal system was substantially attenuated. In addition, LIΔdd:*dal* induced specific CD4^+^/CD8^+^ T‐cell responses and protected mice against LIΔ challenge. Similarly, we constructed an LM balanced‐lethal system LMΔdd:*dal*. Sequential immunization with different recombinant *Listeria* strains will significantly enhance the immunotherapeutic effect. Thus, LIΔdd:*dal* combined with LMΔdd:*dal*, or with other balanced‐lethal systems will be more promising alternative for vaccine development.

## Graphical abstract

The attenuated LI and LM balanced‐lethal stable systems were successfully constructed. LIΔdd:*dal* can proliferate in phagocytic cells and invade non‐phagocytic cells. LIΔdd:*dal* can induce cellular immune responses and enhance the protection against LIΔ.

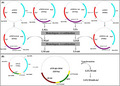

## INTRODUCTION

*Listeria* is widely distributed in the environment (Wang et al., 2017), and only two *Listeria* species, namely, *Listeria monocytogenes* (LM) and *Listeria ivanovii* (LI), are considered pathogenic (Orsi & Wiedmann, 2016). LM is highly pathogenic in both, humans and animals (Mathipa et al., 2019). It can cross the intestinal (Drolia & Bhunia, 2019; Nikitas et al., 2011), blood–brain (Ghosh & Higgins, 2018; Zhang et al., 2018) and foetoplacental barriers (Morrison et al., 2018; Wolfe et al., 2017); thereby leading to severe infections, such as gastroenteritis (Mehmood et al., 2017), meningitis (Koopmans et al., 2017) and septicaemia (Koopmans et al., 2017). Then phagocytosed by antigen‐presenting cells (APCs), such as macrophages, LM escapes the phagocytic vacuoles to enter the cytoplasm (Ruan et al., 2016), thereby enabling the presentation and processing of target antigens by both, the major histocompatibility complex (MHC) class I and II pathways. Antigen presentation by MHC class I and class II results in the subsequent induction of specific CD8^+^ and CD4^+^ T‐cell responses (Wallecha et al., 2009), respectively. The ability of LM to induce both CD8^+^ and CD4^+^ T‐cell responses makes it an attractive candidate as a vaccine vector for specifically delivering viral or tumour‐associated antigens (Flickinger et al., 2018; Miller et al., 2015). Similarly, LI can also multiply in phagocytic and non‐phagocytic cells (Ammendolia et al., 2007; Jiang et al., 2020; Liu, Liu, et al., 2020a; Liu, Tian, et al., 2020b; Lin et al., 2015; Mahdy, Liu, et al., 2019a; Mahdy, Sijing, et al., 2019b).

Although the genome structure, intracellular life cycle and virulence determinants of LI are similar to those of LM, LI mainly infects ruminants (Vázquez‐Boland et al., 2001) and, therefore, exhibits reduced toxicity in humans. Previously, we reported that mice immunization with a recombinant LI strain carrying a *Mycobacterium tuberculosis* antigen in its genome induced an antigen‐specific CD8^+^ T‐cell response that was dominated by interferon‐γ (IFN‐γ) secretion (Lin et al., 2015), confirming that LI could be used as a vaccine vector. Furthermore, a previous study by our group showed that sequential immunization with different recombinant *Listeria* strains significantly enhances the immunotherapeutic effect of *Listeria*‐vector based vaccines. Immunization with *Listeria*‐based recombinant strains (LM and LI) can boost the protective effects of Bacillus Calmette‐Guerin (BCG) against pulmonary mycobacterial infection (Liu, Liu, et al., 2020a; Liu, Tian, et al., 2020b). Additionally, an LI strain carrying the human papillomavirus (HPV)‐E6E7 antigen (LIΔ‐E6E7) as a booster for LMΔ‐E6E7 was found to significantly enhance the anti‐tumour effects of a single‐dose LMΔ‐E6E7 therapy (Su et al., 2021). These studies suggest that both LI and LM hold strong potential as *Listeria*‐vectored vaccines.

The development of live bacterial vectors has provided novel approaches for vaccine construction. The expression level of heterologous antigens is a major concern when developing a live bacterial vector based vaccine. Generally, live bacterial vector vaccines deliver heterologous antigens through multicopy plasmids and use antibiotic resistance genes for screening. However, the multicopy plasmids may be lost rapidly when such strains enter an antibiotic‐free environment, resulting in insufficient expression of the heterologous antigen and thus a reduction in immunogenicity (Galán et al., 1990). Additionally, the antibiotic resistance genes carried by the plasmids may lead to potential safety concerns and increase the burden of drug resistance (Ding et al., 2018). Another method involves directly inserting the pathogen's protective antigen or epitope into the bacterial genome via homologous recombination to ensure that the heterologous protein is expressed stably. However, this approach also requires the use of antibiotic resistance genes for screening. In our previous studies, we integrated heterologous genes into the bacterial genome without introducing antibiotic resistance genes; however, this system had the disadvantage of low expression levels.

These limitations may be overcome by a balanced‐lethal system. A balanced‐lethal system can be defined as a system where a mutated gene, which is necessary for the replication of a nutrient‐deficient strain, is compensated for by a special plasmid carrying the nutrient gene. For a balanced‐lethal system, the screening pressure for the plasmid comes from the survival of the strain rather than from additional antibiotics; and therefore, the plasmid is stably maintained and expressed in the host (Yan et al., 2013). Balance lethal system‐related genes, such as *asd* that encodes the aspartate β‐galactose dehydrogenase, have been reported in gram‐negative bacteria. Strains with deletions in *asd* fail to survive in the absence of exogenous diaminopimelic acid (Galán et al., 1990). Several balanced‐lethal systems based on the *asd* gene have been widely used for *Salmonella* vectored vaccines (Jawale & Lee, 2013; Zhao et al., 2017).

Similarly, *dal* and *dat* genes are two key nutrient genes in LM. The *dal* gene controls the synthesis of alanine racemase (Alr) that converts L‐alanine to D‐alanine, and the *dat* gene encodes D‐amino acid aminotransferase that converts D‐glutamic acid and pyruvate into D‐alanine and α‐ketoglutaric acid. Since D‐alanine is indispensable for the synthesis of the peptidoglycan in bacterial cell walls, deleting both, *dal* and *dat* genes prevents the mutant strain replication in the absence of exogenous D‐alanine (Thompson et al., 1998). The balanced‐lethal systems based on *dal* and *dat* knockout in the LM strain (LMdd) have been developed previously. Amone them, a LMdd strain based therapeutic vaccine was shown to induce tumour‐specific T‐cell responses and prolong the overall survival of osteosarcoma mice model (Mason et al., 2016; Verch et al., 2004). Moreover, since such balanced‐lethal systems do not have antibiotic resistance markers they potentially comply with the Food and Drug Administration regulations and may be further developed for clinical use. However, the functions of the *dal* and *dat* genes in LI have not been clarified, and whether deleting these genes in LI affects bacterial replication remains unknown.

To avoid the spread of an antibiotic resistance marker and address the low expressions of heterologous antigens, we constructed a LI balanced‐lethal system in this research. The *dal* and *dat* genes from the LI‐attenuated strain LIΔ*atcAplcB* were deleted consecutively, and the resulting LIΔdd strain was complemented with the LM *dal* gene via a recombinant plasmid, resulting in a balanced‐lethal system LIΔdd:*dal*. The nutrient‐deficient strain LIΔdd was unable to grow without the addition of D‐alanine, whereas the strain complemented with LM *dal* recovered growth in media without D‐alanine supplementation. To the best of our knowledge, this is the first study to report a balanced‐lethal system in LI. Our results confirm the potential for using the generated balanced‐lethal system as a vaccine vector. We also constructed a recombinant LM strain (LMΔdd:*dal*). Compared to the other relevant reports (Mason et al., 2016; Verch et al., 2004), besides the two nutrient genes, we deleted two major virulence genes in LM, namely, *actA* and *plcB*, making it safer than before. The combined use of LIΔdd:*dal* and LMΔdd:*dal* strains could serve as a powerful platform for constructing safer and more efficient live bacterial vector vaccines in the near future.

## EXPERIMENTAL PROCEDURES

### Bacterial strains and mice

All the strains and plasmids used in this study are listed in Table S1. *Listeria monocytogenes* 10403S and *Listeria ivanovii* PAM55 were provided by Dr. Hao Shen (Department of Microbiology, Perelman School of Medicine, University of Pennsylvania). *Listeria* strains were routinely cultured in brain/heart infusion (BHI) medium (Beijing LuQiao Company) at 37°C. All restriction enzymes were purchased from (NEB England). Erythromycin (Ery; Sigma‐Aldrich) was added at a concentration of 3 μg/ml. D‐alanine (J&K Scientific) was added to the BHI medium at a final concentration of 2 mg/ml (referred to as the D‐BHI plate or broth).

Female C57BL/6 mice, aged 6–8 weeks, were purchased from the Beijing Charles River Animal Laboratory. All mice were maintained under specific‐pathogen‐free (SPF) conditions throughout the experiments at the Animal Center of the School of Public Health at Sichuan University, and the experimental protocols were approved by the Animal Care and Use Committee of Sichuan University.

### Knocking out the *dal* and *dat* genes in *Listeria* and constructing complementation strains

**FIGURE 1:**
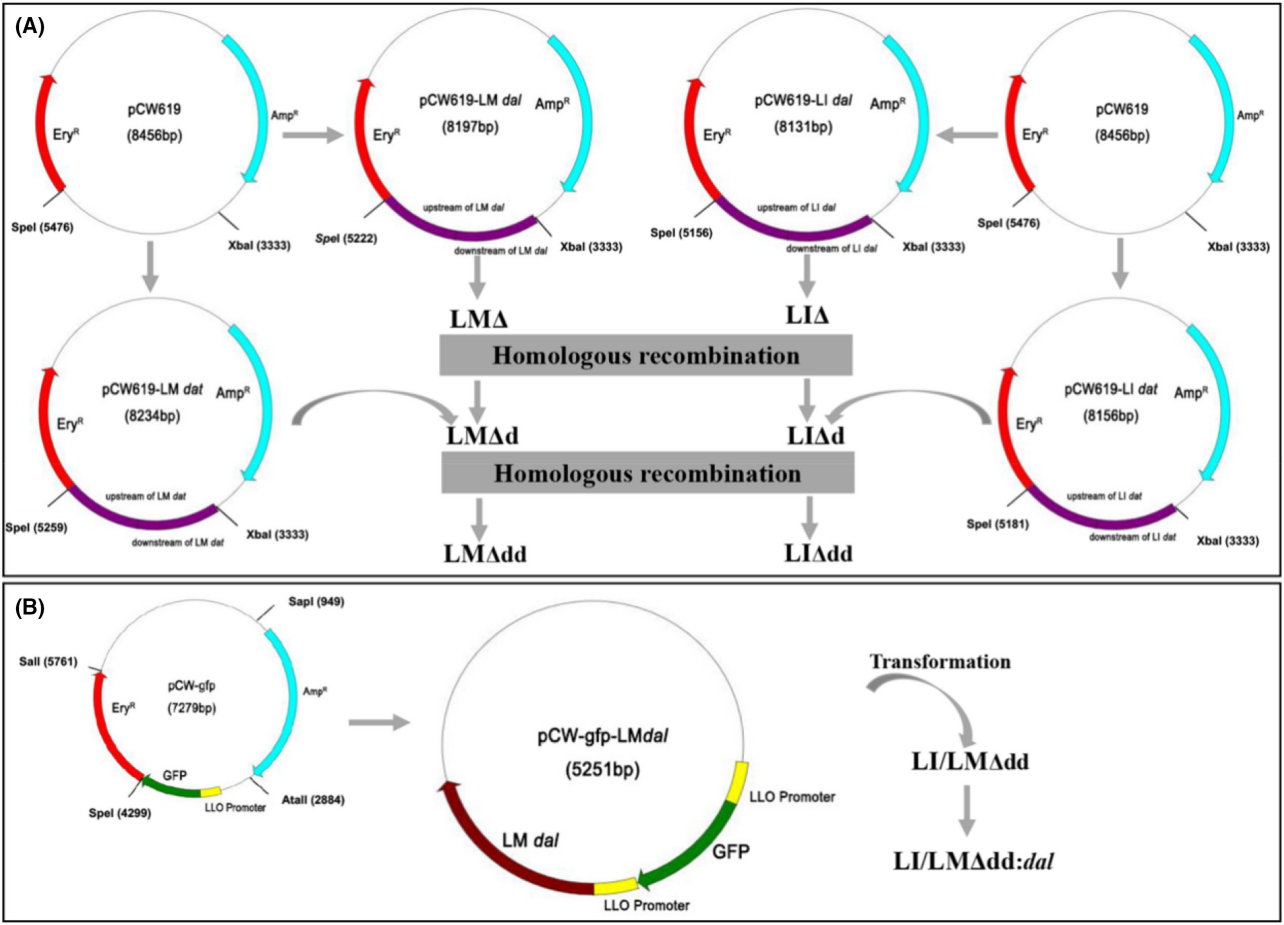
The construction process of the recombinant plasmids and strains. (A) Targeting plasmid pCW619‐LM *dal* and pCW619‐LI *dal*, derived from plasmid pCW619, was transformed into LMΔ and LIΔ, respectively. Then *dal* gene of LMΔ and LIΔ was deleted by homologous recombination. Consequently, the targeting plasmid pCW619‐LM *dat* and pCW619‐LI *dat*, also derived from the plasmid pCW619, was transformed into *dal* gene‐deleted LMΔ and LIΔ, respectively. After homologous recombination, the *dat* gene of the strains was deleted, thus LMΔdd and LIΔdd were constructed. (B) Antibiotic resistance‐free pCW‐GFP‐LM *dal* plasmid, derived from plasmid pCW‐GFP, which harboured LM *dal* gene under the control of LLO promoter, was transformed into LMΔdd and LIΔdd, resulting in LMΔdd:*dal* and LIΔdd:*dal*.

Genes in the LM and LI‐attenuated strains (LMΔ and LIΔ) were knocked out as described previously (Wang et al., 2014). Four DNA fragments, the upstream and the downstream fragments of the LM *dal* gene (984 and 930 bp, respectively) and of the LM *dat* gene (959 bp and 989, respectively) were amplified by polymerase chain reaction (PCR). Another 4 DNA fragments, the upstream and the downstream fragments of the LI *dal* gene (991 bp and 857 bp, respectively) and of the LI *dat* gene (970 and 899 bp, respectively) were also amplified by PCR. The corresponding upstream and downstream homologous arms were fused by overlap PCR. The resulted upstream‐downstream fused fragments contained complementary sequences of both the terminal ends of the XbaI‐ and SpeI‐digested linearized plasmid pCW619 (NCBI accession no. MN513049). The PCR products were cloned into the XbaI and SpeI site in the pCW619 by an In‐Fusion cloning kit (Tsingke Company, China), resulting in the pCW619‐LM *dal* (NCBI accession no. MN528127), pCW619‐LM *dat* (NCBI accession no. MN 528128), pCW619‐LI *dal* (NCBI accession no. MN528129) and pCW619‐LI *dat* (NCBI accession no. MN528130) plasmids. After verification by PCR screening, restriction analysis and sequencing, the target plasmids, namely, pCW619‐LM *dal* and pCW619‐LI *dal* were electroporated into the corresponding competent LMΔ or LIΔ cells. Presumptively positive colonies were selected on erythromycin (3 μg/ml) supplemented BHI plates and confirmed by colony PCR. Positive colonies were grown on erythromycin (3 μg/ml) supplemented BHI plates at 42°C for 2 to 3 generations. Putative gene swapping positive colonies were subsequently grown in BHI broth at 30°C for six consecutive passages. Deletion of the *dal* gene was confirmed using PCR and gene sequencing. Similarly, pCW619‐LM *dat* and pCW619‐LI *dat* were introduced into the corresponding *dal* gene‐deleted strains to obtain LMΔdd and LIΔdd (Figure 1A).

To construct the complementation strains, the listeriolysin (LLO) promoter (Zhang et al., 2017), that is *phly* and LM *dal* gene (NCBI accession no. AEO05885.1) in LM were amplified by PCR and fused by overlap PCR. Then the products were cloned into the SpeI and SalI site of the pCW‐GFP vector to replace the Ery^R^ fragment, resulting in the pCW‐GFP‐LM *dal* plasmid. Next, pCW‐GFP‐LM *dal* was digested with SapI and AtaII to delete the Amp^R^ fragment and ligated using a DNA blunting kit (TaKaRa Biotechnology). After ethanol precipitation, the antibiotic resistance‐free plasmid pCW‐GFP‐LM *dal* (NCBI accession no. MN513050) was transformed into LMΔdd or LIΔdd, resulting in the LMΔdd:*dal* and LIΔdd:*dal* strains, respectively (Figure 1B).

### Growth curve analysis

LMΔdd and LIΔdd were routinely grown overnight in D‐BHI broth at 37°C. LMΔdd:*dal* and LIΔdd:*dal* were cultivated in BHI broth at 37°C and 200 rpm. Thereafter, 3 ml of the overnight cultures were added to 50 ml of BHI broth or BHI broth containing a certain concentration of D‐alanine (200 μg/ml). The absorbance of the broth was adjusted to an initial value of 0.06 at 600 nm (*A*_600_) by using Multiskan Go Microplate Reader (Thermo Fisher Scientific). The bacterial cultures were incubated at 37°C and 200 rpm, on a shaker. Samples were collected every hour for *A*_600_ measurements.

### In vitro stability of the complementation strains

To examine whether the LMΔdd:*dal* and LIΔdd:*dal* strains could grow stably in vitro, 1 μl of the overnight cultures were added to 5 ml BHI broth and incubated at 37°C for 25 consecutive passages. Bacteria cultured at 37°C for 16 h was considered as one generation. Bacterial samples from the 5, 10, 15, 20 and 25th generations were collected to assess the stability of the bacterial genes by PCR. Primers used were as follow: specific primers for LM (LM‐orfBAldh; F:5′‐ CTT CGA TGA CAA CAG CTG TAC C‐3′; R: 5′‐AAT CCT AAA GCA TGC GCC TTC G‐3′), specific primers for LI (LI‐orfBAldh; F:5′‐GCA TGC TTT TAA GAT GAA GTC TCA C‐3′; R: 5′‐CAA AAA ATC ATT TTA GTT GGC GAC GG‐3′), LMdal‐F/R (F:5′‐GAA CAA AAT AAA CTC CCG GAA‐3′; R: 5′‐TAC TTT TGA ACC AGT TTG AAA T‐3′), HomoLMdal‐F/R (F:5′‐CTT ACG AAA TTT CTT TTG CAG‐3′; R: 5′‐GTT GAT ACG CGT TGT TTG TA‐3′), HomoLIdal‐F/R (F: 5′‐CGG GCT AGA AGC TTT ACC A‐3′; R: 5′‐GGT TTC TGA TGT CCC TTA AAT C‐3′), HomoLMdat‐F/R (F:5′‐GCT TGT ACC ATA CGC CCA G‐3′; R: 5′‐CAA TCA TTC ACG CGG AAA A‐3′) and HomoLIdat‐F/R (F: 5′‐GTT TGT TTT CCT GCT TG‐3′; R: 5′‐TTT GCC ACT TTC TCG T‐3′).

### Characterization of the biochemical property

Strains were inoculated in various sugar fermentation tubes using biochemical test kits (Beijing LuQiao Company). Parallel double tubes were used for each experiment for the nutrient‐deficient strain. A final concentration of 400 μg/ml of D‐alanine was used for LIΔdd and 100 μg/ml of D‐alanine for LMΔdd.

### Cell lines

The murine macrophage RAW264.7 and murine hepatocellular carcinoma Hepa1‐6 cell lines were obtained from the Cells Bank of the Chinese Academy of Sciences. RAW264.7 and Hepa1‐6 cells were cultured in complete Dulbecco's modified Eagle's medium (DMEM; Gibco) supplemented with 10% foetal bovine serum (FBS; GIBCO), 100 U penicillin (Sigma) and 100 μg/ml streptomycin (Sigma). The cells were incubated at 37°C in a humidified 5% CO2 incubator and subcultured at a confluence of 80%–90%.

### Bacterial replication in RAW264.7 cells

RAW264.7 cells were cultured in 24‐well plates to obtain 90% confluent monolayers (1 × 10^6^ cells/ml). LIΔ, LIΔdd and LIΔdd:*dal* were cultured until the stationary phase was reached. The bacterial cells were collected and added to the RAW264.7 cells at a multiplicity of infection (MOI) of 20:1. After 1 h of infection, all wells were washed twice with PBS, and DMEM containing 200 μg/ml gentamycin was added to kill the bacteria that had failed to enter the cells. After 1 h of incubation, 0.1% Triton X‐100 (Solarbio) was added to two of the wells to lyse the cells and the intracellular bacteria were enumerated by plating. The other wells were washed twice with PBS, and DMEM containing 20 μg/ml gentamicin was added for subsequent incubation until to be determined the intracellular bacteria by plating after 4, 6 and 8 h of infection.

LIΔ and LIΔdd expressed the green fluorescent protein (GFP) after transformation with the pCW‐GFP plasmid. Next, the RAW264.7 cells were infected with the GFP labelled strains or LIΔdd:*dal* according to the steps above. The RAW264.7 cells were collected at 2 and 6 h post‐infection. Thereafter, 0.2 ml 4′,6‐diamidino‐2‐phenylindole (DAPI; Solarbio) was added to each well and the cells were incubated at 25 ± 2°C for 5 min for nuclear staining. Visualization was performed using a laser scanning confocal microscope (LSCM) (Zeiss).

### Cellular adhesion and invasion assays

Adhesion and invasion assays were performed using the Hepa1‐6 cells. Moreover, 90% confluent monolayers (1 × 10^6^ cells/ml) of Hepa1‐6 cells in 24‐well plates were inoculated with bacteria from the exponential phase at an MOI of 10:1. For the adhesion assays, loosely bound bacteria were removed from the cells by washing with phosphate buffered saline (PBS; Solarbio) after infection for 1 h. The Hepa1‐6 cells were then lysed with 0.1% Triton X‐100, 10‐fold diluted and plated on BHI or D‐BHI plates to count the viable adherent bacteria. For the invasion assays, after 1 h of infection with bacteria from the exponential phase, each well was washed, and fresh medium containing 200 μg/ml gentamycin was added and the cells were incubated at 37°C for 1 h. The cells were then lysed with 0.1% Triton X‐100 to count viable intracellular bacteria. The adhesion and invasion efficiency were expressed as the percentage of the bacterial CFU that adhered to or invaded cells vs. bacteria CFU inoculated.

### Virulence of the *Listeria* strains

The 50% lethal dose (LD50) was determined in 6 to 8 weeks old female C57BL/6J mice. Mice were intravenously injected with 0.1 ml graded doses (8 × 10^8^, 3 × 10^9^ and 5 × 10^9^ CFU/ml) of LIΔdd, LMΔdd, LIΔdd:*dal* and LMΔdd:*dal* (10 mice/group). The mice were monitored for 10 days.

### In vivo distribution of the complementation strain post intravenous injection

Mice were intravenously injected with LIΔdd:*dal* at the maximal safe dose of 5 × 10^8^ CFU. The level of infection in each mouse in the liver and spleen was determined by organ harvesting, homogenizing, diluting and plating for CFU (Hanson et al., 2019). Briefly, spleen and liver of mice were mixed with 400 μl and 1 ml of 0.1% Triton X‐100 solution, respectively, homogenized and were 10‐fold serially diluted in saline solution, and then 20 μl of homogenate or dilutions were plated on BHI and D‐BHI agar plates. Viable bacterial loads in the liver and spleen were determined at 1, 2, 3, 5, 7 and 14 days post‐inoculation (dpi) by plating on BHI or D‐BHI plates. The detection limits of this procedure were 20 CFU/spleen and 50 CFU/liver. For measurement of the gene stability of LIΔdd:*dal* in liver and spleen, bacteria were recovered from the spleen and liver of infected mice at 2 and 5 dpi with BHI and D‐BHI plates, respectively. Three colonies recovered from each mouse by BHI and D‐BHI plates were randomly selected to verify the stability of plasmids and the stably deletion of *dal*/*dat* genes by colony PCR using the same primers as mentioned above and sequencing (Tsingke Biotechnology Co., Ltd).

### Antigen‐specific cytokine assay

For the cytokine assay, 6–8 weeks old C57BL/6 mice were randomly divided into three groups (10 mice/group) and intravenously immunized twice at an interval of 7 days with 2 × 10^8^ CFU LIΔ, 5 × 10^8^ CFU LIΔdd:*dal*, or 100 μl PBS. On the 9th day, after the second immunization, the spleens of C57BL/6 mice were removed and made into single‐cell suspensions. Erythrocytes were lysed by ammonium chloride and splenocytes were washed, counted and added to round‐bottom 96‐well microplates at a concentration of 2.5 × 10^6^ cells per well. Then the cells were stimulated in the presence of GolgiStop (BD PharMingen) with 5 μg/ml ILO protein for 5 h and incubated at 37°C. Staining was performed using FITC rat anti‐mouse CD3, PerCP rat anti‐mouse CD4, APC‐CyTM7 Rat Anti‐Mouse‐CD8a (BD PharMingen), PE rat anti‐mouse interferon‐γ (IFN‐γ), PE‐CyTM7 rat anti‐mouse tumour necrosis factor‐α (TNF‐α) and APC rat anti‐mouse IL‐2 (BioLegend). The cells were fixed using the Cytofix/Cytoperm Kit (BD PharMingen). The cells were acquired on a BD FACSverse flow cytometer (BD Biosciences) and analysed using the FlowJo 7.6 software (BD Biosciences).

### Challenge test with LIΔ

C57BL/6 mice were immunized via the tail vein as described above. The mice were challenged intravenously with LIΔ (5 × LD50, 1 × 10^9^ CFU) one week after the second immunization. Disease symptoms, mortality and body weight was monitored daily for 9 days.

### Statistical analysis

The experimental data were analysed by SPSS21.0, Data on bacterial growth was compared using the LSD method, and all other data were analysed by the Kruskal‐Wallis nonparametric test or Brown‐Forsythe and Welch Analysis of Variance (ANOVA) tests. *p* < 0.05 was considered significant.

## RESULTS

### In vitro growth characteristics of *dal*/*dat* double knock out strains and *dal* gene complementation strains

**FIGURE 2:**
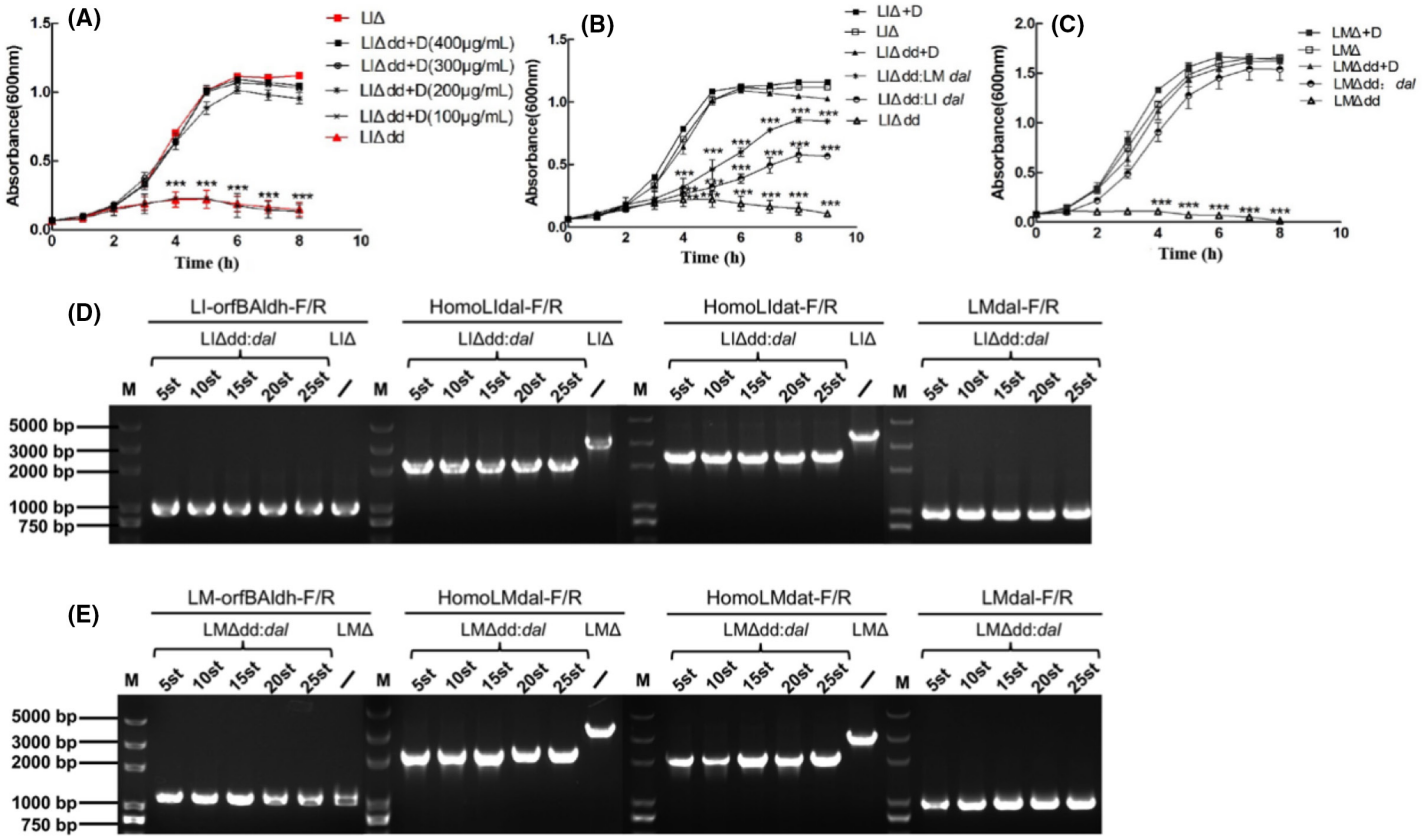
Growth curve and genetic stability of constructed strains. (A) LIΔdd were grown in BHI broth in the presence of gradually increased concentration of D‐alanine (100, 200, 300 and 400 μg/ml) at 37°C. (B) LIΔdd and LIΔ were grown in BHI broth in the presence or absence of exogenous D‐alanine (400 μg/ml) at 37°C. LIΔdd:*dal* grown in ordinary BHI broth at 37°C. (C) LMΔdd and LMΔ were grown in BHI broth in the presence or absence of exogenous D‐alanine (100 μg/ml) at 37°C. LMΔdd:*dal* grown in ordinary BHI broth at 37°C. Three independent experiments were repeated. Data were accumulated from three experiments and expressed as means ± SEM. *p* values were determined by LSD method. *p < 0.05; **p < 0.01 and ***p < 0.001 were not marked in growth curve. (D) The lengths of PCR products of the 5, 10, 15, 20 and 25st generation of LIΔdd:*dal* amplified by primers LI‐orfBAldh‐F/R, HomoLIdal‐F/R, HomoLIdat‐F/R and LMdal‐F/R were as expected. (E) The lengths of PCR products of the 5, 10, 15, 20 and 25st generation of LMΔdd:*dal* amplified by primers LM‐orfBAldh‐F/R, HomoLMdal‐F/R, HomoLMdat‐F/R and LMdal‐F/R were as expected.

The *dal/dat* double mutant strains were successfully screened after homologous recombination. A description of the construction process for the recombinant strains is shown in Figure 1. Gene sequencing confirmed that the *dal* (1107 bp) and *dat* genes (870 bp) were deleted from the chromosomes of LMΔ and LIΔ. The *dal/dat* double knockout LM and LI strains required D‐alanine supplemented BHI medium for growth. Figure 2A–C shows that LMΔdd required a growth medium supplemented with 100 μg/ml D‐alanine for in vitro growth, whereas LIΔdd required a growth medium supplemented with at least 200 μg/ml D‐alanine. The growth rate of LIΔdd increased to the degree of LIΔ at a concentration of 400 μg/ml D‐alanine. Therefore, all subsequent experiments for LIΔdd were performed using medium supplemented with 400 μg/ml of exogenous D‐alanine.

To develop a balanced‐lethal system in the LMΔdd and LIΔdd strains, a complement plasmid (pCW‐GFP‐LM *dal*) was constructed. Specifically, the Ery^R^ fragment of the pCW‐GFP plasmid was replaced with the LM *dal* gene fused to the LLO promoter. The Amp^R^ fragment in the plasmid was deleted by *Sap*I and *Ata*II double enzyme digestion. With the antibiotic resistance‐free plasmids transformed, the nutrient‐deficient strains recovered growth capacity on BHI medium without D‐alanine supplementation (Figure 2B,C). Unexpectedly, complementation with the LI *dal* gene was not as effective as complementation with the LM *dal* gene in restoring the growth of LIΔdd.

### In vitro genetic stability of the balanced‐lethal system

To examine the in vitro genetic stability of the balanced‐lethal system, LIΔdd:*dal* and LMΔdd:*dal* were passaged in BHI broth for 25 generations. Primers (LI‐orfBAldh‐F/R, HomoLIdal‐F/R and HomoLIdat‐F/R) were designed for PCR to obtain the corresponding 954, 3158 and 3188 bp length products from LIΔ. Primers (LM‐orfBAldh‐F/R, HomoLMdal‐F/R and HomoLMdat‐F/R) were designed for PCR to obtain the corresponding 1012, 3083 and 3248 bp length products from LMΔ. Since the *dal* gene (1107 bp) was deleted from the chromosomes of LIΔ and LMΔ, the corresponding amplification products were expected to be 2051 and 1976 bp, respectively. The *dat* gene was deleted from the chromosomes of LIΔ and LMΔ, and the corresponding amplification products were expected to be 2221 and 2141 bp, respectively. The 5, 10, 15, 20 and 25th generations of LIΔdd:*dal* or LMΔdd:*dal* were collected for amplification, and the lengths of PCR products were as expected, showing that the nutrient‐deficient strains were genetically stable (Figure 2D,E). We then used the LMdal‐F/R primers to detect the LM *dal* gene in the complement plasmid. The expected 909 bp product was amplified from the 5, 10, 15, 20 and 25th generations of the LIΔdd:*dal* or LMΔdd:*dal* strains (Figure 2D,E), indicating that the complement plasmid was stably present within the attenuated strains. Taken together, the LI and LM balanced‐lethal systems were stable and heritable.

### Toxicity in mice

The LD50 (C57BL/6 mice, i.v.) of the LI‐attenuated strains (LIΔ) was about 10^8^ CFU, LD50 of LI wild‐type strain was about 10^6^ CFU (Lin et al., 2015). The LD50 of LMΔ was approximately 5 × 10^7^ (Zhou et al., 2016). To determine the LD50 of the recombinant strains, groups of mice were injected with graded doses of LMΔdd, LIΔdd, LMΔdd:*dal* or LIΔdd:*dal*. According to the pre‐experiments results and our previous animal experiment experience, when mice were intravenously inoculated with a *Listeria* strain suspension whose concentration is greater than 10^9^ CFU, the mice will die within 30 minutes after inoculation (data not shown). Such acute death is considered to be unrelated to the bacteria infection. Therefore, the highest dose was set to 5 × 10^8^ CFU. Ten days post‐injection, we observed that all mice from the LMΔdd, LIΔdd, LMΔdd:*dal* and LIΔdd:*dal* groups survived, whereas only 20% of mice from the highest dose group in LMΔdd:*dal* failed to survive. Therefore, the LD50 of LMΔdd, LIΔdd and LIΔdd:*dal* was higher than 5 × 10^8^ CFU, and that of LMΔdd:*dal* was higher than 3 × 10^8^ CFU, indicating that these strains were notably attenuated compared to the parent strains. The doses of LIΔdd and LIΔdd:*dal* were set at 5 × 10^8^ CFU for all subsequent animal experiments. The inoculation dose and mouse mortality rates are listed in Table S2.

### Biochemical properties of the constructed strains

To investigate the effect of knocking out the *dal* and *dat* genes on the metabolic ability of the strain, the biochemical characteristics of the strains were tested. Table 1 shows that for all tested sugars except xylose were identical for the LIΔdd and parent strains after alanine supplementation. The sugar fermentation results for LIΔdd:*dal* were consistent with that of the parent strain for all tested sugars except xylose. After alanine supplementation or *dat* gene complementation, the sugar fermentation results of LMΔdd were consistent with LMΔ.

**TABLE 1.** Biochemical characteristics of *Listeria* balanced‐lethal system

Strains	Biochemical characteristics					
	Glu	Mal	Man	Rha	Xyl	Esc
LIΔ	+	−	−	−	+	+
LIΔdd+D	+	−	−	−	−	+
LIΔdd:*dal*	+	−	−	−	−	+
LMΔ	+	+	−	+	−	+
LMΔdd+D	+	+	−	+	−	+
LMΔdd:*dal*	+	+	−	+	−	+

Abbreviations: +, positive; −, negative; Esc, ‐Esculin; Glu, Glucose; Mal, Maltose; Man, Mannitol; Rha, Rhamnose; Xyl, Xylose.

### Replication, adhesion and invasion ability of LI balanced‐lethal system

**FIGURE 3:**
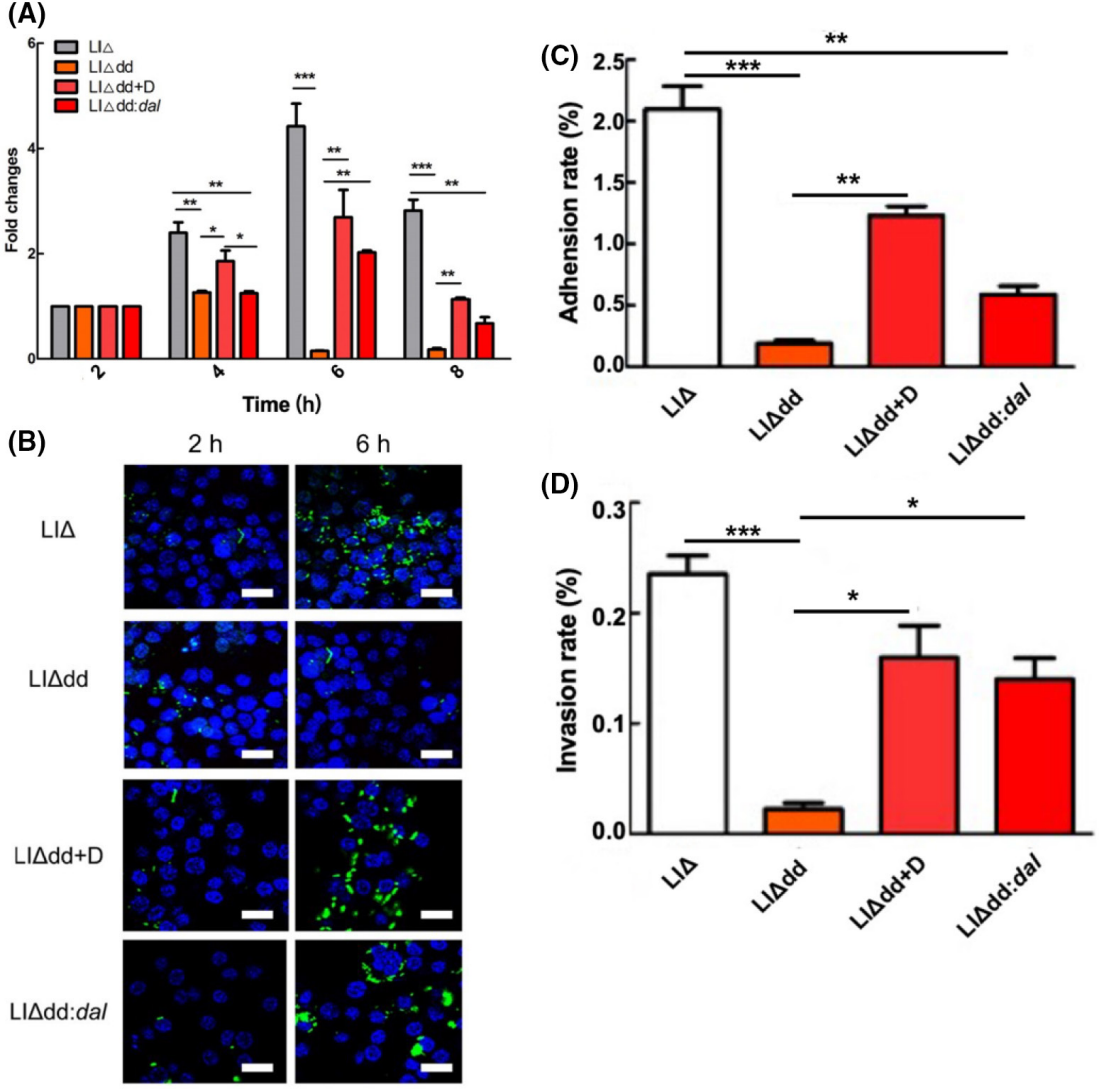
The abilities of proliferation, adhesion and invasion to cells. (A) Intracellular proliferation of LIΔ, LIΔdd, LIΔdd+D (400 μg/ml of D‐alanine) and LIΔdd:*dal* in RAW264.7 cells at an MOI of 20:1. Intracellular proliferation was expressed by fold changes (*Y* axis). Fold change was calculated as: number of intracellular bacteria at each time point vs. number of bacteria invaded in cells (number of intracellular bacteria at 2 h post infection). (B) LSCM imaging to show the intracellular growth of the strains in RAW264.7 cells (MOI was 20:1). Green: GFP‐expressing bacteria; Blue: DAPI‐stained cell nucleus; The scale bar in images were 20 μm. (C) The adhesion ability of LIΔ, LIΔdd, LIΔdd+D (400 μg/ml of D‐alanine) and LIΔdd:*dal* to Hepa1‐6 cells at a MOI of 10:1. Adhesion rate was expressed as the percentage of the bacteria adhered to Hepa1‐6 cells vs. inoculated bacteria at 1 h after infection. (D) The invasion ability of LIΔ, LIΔdd, LIΔdd+D (400 μg/ml of D‐alanine) and LIΔdd:*dal* to Hepa1‐6 cells at a MOI of 10:1. Invasion rate was expressed as the percentage of the intracellular bacteria 1 h post gentamycin treatment vs. initial inoculated bacteria. The box plots depict mean ± SEM and the whiskers min‐max values from more than three independent experiments. *p < 0.05; **p < 0.01 and ***p < 0.001 (Kruskal–Wallis test).

*Listeria* can adhere to and invade many types of cells and survive in macrophages and non‐phagocytic cells, such as hepatocytes, epithelial cells and endothelial cells (Vázquez‐Boland et al., 2001). To investigate if LIΔdd could replicate in macrophages and if its proliferation capacity increased after complementing the *dal* gene, intracellular bacterial CFUs were determined at various time points after infection. The intracellular replication curve showed that both LIΔ and LIΔdd:*dal* proliferated in macrophages, but the proliferative capacity of LIΔdd:*dal* is not fully restored compared to LIΔ (Figure 3A). The number of intracellular bacteria peaked at 6 h post‐infection and gradually decreased thereafter. LIΔdd showed poor replication capacity in RAW264.7 cells. After D‐alanine supplementation during infection, LIΔdd showed the same proliferation tendency as the parent strains. The results showed that knocking out the *dal* and *dat* genes reduced the ability of LI to proliferate in macrophages, and the proliferation ability to proliferate could be partly restored after *dal* gene complementation.

We also observed intracellular proliferation using LSCM (Figure 3B). LIΔ and LIΔdd were labelled with GFP using the pCW‐GFP plasmid, and LIΔdd:*dal* was labelled with GFP using the complement plasmid pCW‐GFP‐LM *dal*. The intracellular fluorescence intensity was significantly higher at 6 h post‐infection than that at 2 h, indicating that LIΔ and LIΔdd:*dal* could proliferate in the macrophages. However, the intracellular fluorescence intensity in the nutrient‐deficient strain at 6 h post‐infection did not differ significantly from that at 2 h, indicating that LIΔdd could not proliferate in macrophages. The confocal microscopy data were consistent with the CFU results of intracellular bacteria.

Adhesion and invasion assays were performed by infecting Hepa1‐6 cells at an MOI of approximately 10:1 (Figure 3C,D). After 1 h of infection, we observed that the adhesion rate of LIΔdd (0.19%) was significantly lower than that of LIΔ (2.1%). The adhesion rate of LIΔdd increased to 1.2% or 0.59% after D‐alanine supplementation or *dal* gene complementation, respectively. The ability of LIΔdd:*dal* to invade Hepa1‐6 improved after complementation with *dal* gene. These results suggest that knocking out the *dal* and *dat* genes reduced the ability of LIΔ to adhere and invade Hepa1‐6 cells. Finally, *dal* gene complementation restored the ability of the LIΔdd strain to adhere and invade cells.

### In vivo growth characteristics and stability of the LI balanced‐lethal system

**FIGURE 4:**
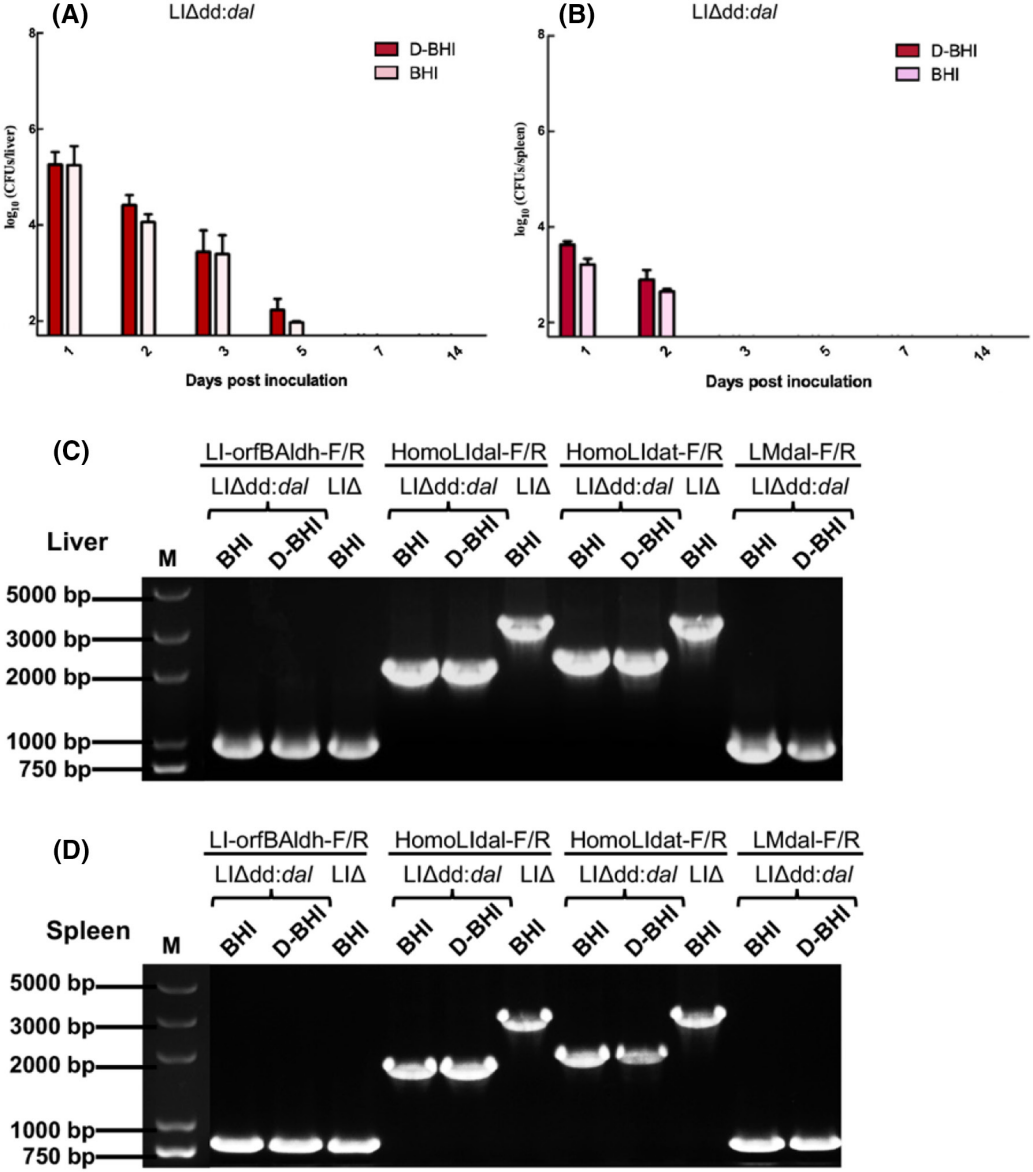
The growth curve and genetic stability of LIΔdd:*dal* in vivo in mice liver and spleen. Groups (36 mice in each) of C57BL/6J mice were injected in the tail vein with maximum safe dose of LIΔdd:*dal* (5 × 10^8^ CFU), and six mice of each group were sacrificed at 1, 2, 3, 5, 7 and 14 dpi. The CFU in the mice liver or spleen was determined by plating on both BHI and D‐BHI plates. (A) The CFU in the mice liver. (B) The CFU in the mice spleen. Results were expressed as means ± SEM per group. *p* values were determined by Kruskal‐Wallis test. The detection limit was 20 CFU/spleen and 50 CFU/liver. (C) The PCR products of LIΔdd:*dal* isolated from mouse liver that amplified by primers LI‐orfBAldh‐F/R, HomoLIdal‐F/R, HomoLIdat‐F/R and LMdal‐F/R. (D) The PCR products of LIΔdd:dal isolated from mouse spleen that amplified by primers LI‐orfBAldh‐F/R, HomoLIdal‐F/R, HomoLIdat‐F/R and LM‐F/R.

The in vivo growth curve was obtained by examining the number of viable bacteria in the homogenized spleens and livers of LIΔdd*:dal*‐immunized C57BL/6 mice at 1, 2, 3, 5, 7 and 14 days post‐infection. We found that LIΔdd*:dal* was more likely to colonize the liver (Figure 4). At 1‐day post infection, the number of LIΔdd:*dal* reached 10^5^ CFU in the livers of mice and then decreased gradually. The bacterial count in the liver decreased below the detection limit at 7 days after infection, indicating that the bacteria were cleared from the liver. The number of LIΔdd:*dal* in the spleens of mice reached 10^3^ CFU at 1 day post infection and then decreased gradually. At 3 days post infection, bacteria could not be detected in the spleen. Bacterial CFU on both plates did not differ significantly (*p* > 0.05).

The in vivo stability of the LI balanced‐lethal system was further determined by PCR and sequencing. Bacterial colonies on both plates were randomly selected for PCR using primers LI‐orfBAldh‐F/R, HomoLIdal‐F/R, HomoLIdat‐F/R and LMdal‐F/R. The lengths of the PCR products from colonies on both plates were the same, were 954, 2051, 2221 and 909 bp as expected (Figure 4C,D). In addition, the sequencing results of the PCR products of LIΔdd:*dal* that recovered from mice liver and spleen indicated the complete deletion of the *dal* and *dat* gene without mutations (Figure S1A,B), and the pCW‐GFP‐LM *dal* stably existed in LIΔdd:*dal* (Figure S1C).

### Specific cellular immune response to LIΔ and LIΔdd:*dal*

**FIGURE 5:**
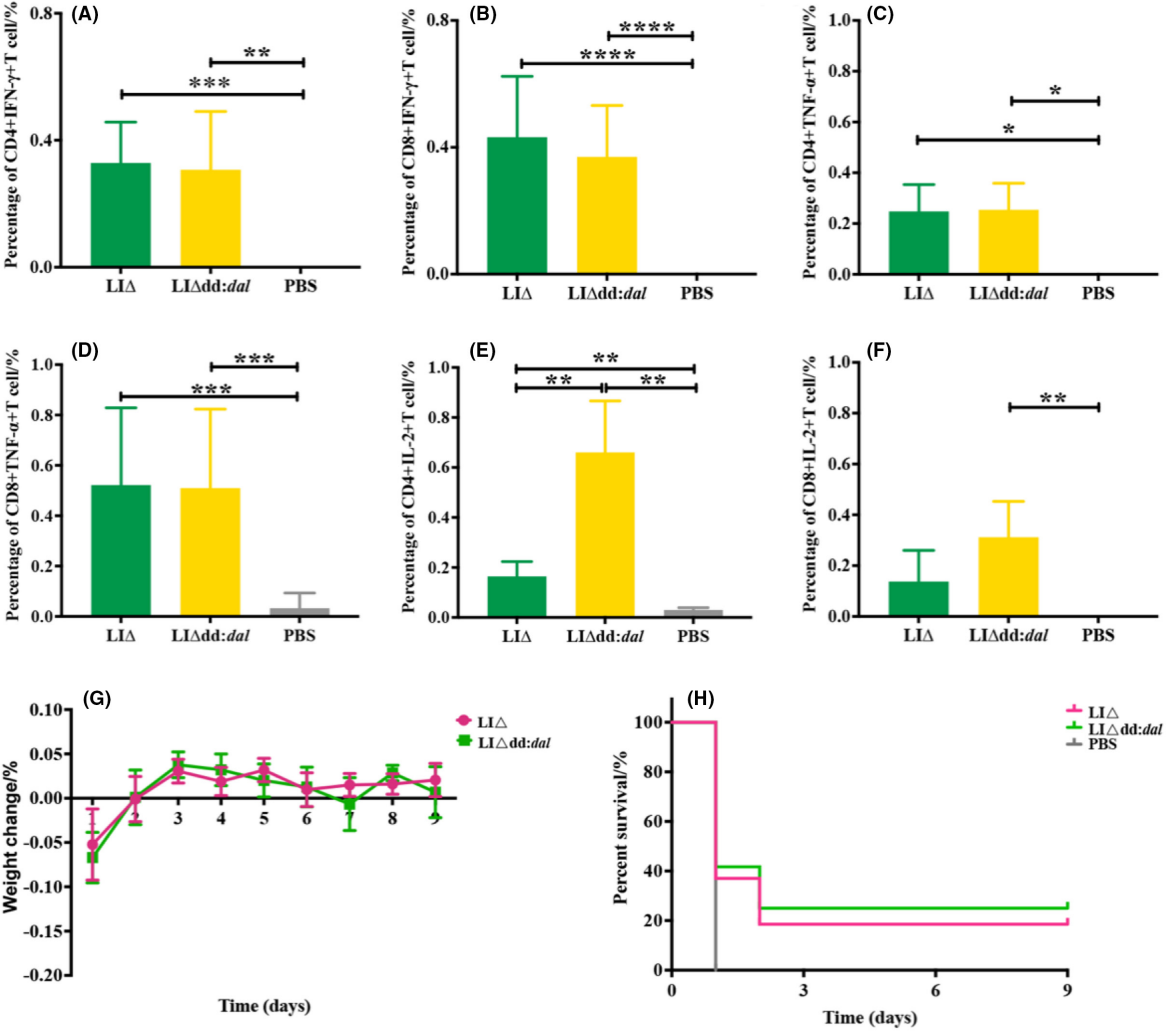
ILO‐specific T cells cytokines in the mice spleen. (A, B) Percentages of IFN‐γ‐secreting cells in the T cell. (C, D) Percentages of TNF‐α‐secreting cells in the T cell. (E, F) Percentages of IL‐2‐secreting cells in the T cell. (G) Daily change in body weight of immunized mice after LI strains infection. (H) Survival rate of immunized mice infected with LI strains. Data were expressed as means ± SEM per group of 10 mice. *p < 0.05 (Kruskal–Wallis test).

We successfully expressed the ILO protein in an *E. coli* based expression system and then examined ILO‐specific cellular immune responses induced by LIΔ and LIΔdd:*dal* (Figure 5A–F). The levels of cytokines secreted by T cells were significantly higher in LIΔ‐ and LIΔdd:*dal*‐immunized mice than that in mice injected with the PBS control. Interestingly, the proportion of IL‐2 secreting CD8^+^ T cells was significantly higher in LIΔdd:*dal* vaccinated animals than in PBS‐vaccinated mice (*p* < 0.05), whereas there was no difference in levels of IL‐2 secreting CD8^+^ T cells in LIΔ‐immunized and PBS treated mice (Figure 5F). Moreover, mice vaccinated with LIΔdd:*dal* showed a significantly higher proportion of IL‐2 cytokine‐secreting CD4^+^ T cells than LIΔ vaccinated mice (*p* < 0.05, Figure 5E). Our results showed that LIΔdd:*dal* induced stronger antigen‐specific T‐cell immune responses than LIΔ.

### Immune protection efficacy of the LI balanced‐lethal system

To evaluate the efficacy of the immune protection provided by LIΔdd:*dal*, we challenged LIΔdd:*dal*‐immunized mice with LIΔ. We observed that compared to treatment with the PBS control, LIΔdd:*dal* and LIΔ immunization significantly increased the survival rates of mice and enhanced immune protection against pathogenic LIΔ. Moreover, greater protection was observed in the LIΔdd:*dal*‐immunized group (25%) than in the LI immunized group (20%) following challenge with LIΔ infection (Figure 5G). Although the body weight of LIΔdd:*dal‐*immunized mice decreased significantly after challenge, it returned to normal body weight two days later (Figure 5H).

## DISCUSSION

The balanced‐lethal system is based on nutrient genes, which are necessary for bacterial survival. Moreover, live bacterial vectors are important for vaccine development. The balanced‐lethal system described here not only solves the biosafety problem caused by plasmids, such as antibiotic resistance gene transmission, but also solves the problem of unstable passage of the plasmid in the host. In most gram‐positive bacteria, deletion of the genes that control Alr synthesis affects the supply of D‐alanine to the organism, leading to the death of bacteria (Tanner, 2002). However, LM can still survive after deleting the Alr‐encoding *dal* gene because it has a bypass way to synthesize D‐alanine. That is, the *dat* gene, which encodes alanine aminotransferase. In this way, D‐glutamic and pyruvic acid are the resources to synthesize D‐alanine and α‐ketoglutaric acid. Therefore, LM fails to survive in a medium that is not supplemented with D‐alanine only when both, *dal* and *dat* genes are deleted (Thompson et al., 1998). To date, there have been no reports on the mechanism underlying D‐alanine synthesis in LI. We hypothesized that the pathways mediating D‐alanine synthesis in LI would resemble those in LM. Our growth curve analysis confirmed the hypothesis that the LI strain could not grow in the absence of exogenous alanine following deletion of the *dal* and *dat* genes. In addition, we found that LIΔdd was more dependent than LMΔdd on the D‐alanine supplementation. The results showed that LMΔdd could grow in medium containing 100 μg/ml alanine, whereas LIΔdd could only grow when the medium was supplemented with at least 200 μg/ml alanine. Complementation with the LI *dal* gene was not as effective as complementation with the LM *dal* gene in restoring the growth of LIΔdd. However, the mechanism underlying this observation remains to be elucidated.

The complementary plasmid was constructed on the pCW‐GFP backbone. The nutrient‐deficient strains harbouring the complement plasmid could grow in BHI medium, indicating that the *dal* gene on the complement plasmid was successfully expressed in vitro. Strains harbouring the complement plasmid also expressed GFP. In the future, when using this system as a vaccine vector, the *gfp* gene in the plasmid could be replaced by an exogenous antigen gene to accomplish antigen expression. The passage test confirmed the genetic stability of this system in vitro. We then tested its genetic stability in vivo. Bacteria from the livers and spleens of mice inoculated with LIΔdd:*dal* were recovered on both BHI and D‐BHI plates. While only bacteria carrying the complement plasmid were recovered on the BHI plate, those on the D‐BHI plate represented the total number of strains with or without plasmid. We observed no difference in the number of bacteria grown on either plate and in the colony PCR and sequencing results, indicating that LIΔdd:*dal* stably carried the plasmid in vivo.

Most bacteria catalyse xylulose from xylose using a xylose isomerase (XI) that is encoded by the *xylA* gene. The xylulose is then catalysed by the pentose phosphate pathway (HMP) (Moysés et al., 2016). Enzymes and genes related to xylose metabolism have not been reported in *Listeria*. The sugar fermentation tests revealed that LIΔdd could not metabolize xylose, even after alanine supplementation or *dal* gene complementation. Therefore, we speculate that the deletion of *dal* and *dat* genes affects the expression of *xylA* gene and thus the metabolism of xylulose in LI, which highlights the importance for exploring the additional functions of *dal* or *dat* genes in LI.

*Listeria* can survive in phagocytic cells. After being engulfed by phagocytic cells, the bacteria can escape the phagocytic vacuoles, enter the cytoplasm, proliferate and then infect adjacent cells. This property makes *Listeria* a good tool for presenting antigens to lymphocytes, enabling the host to produce an efficient antigen‐specific cellular immune response. We tested the intracellular proliferative ability of LIΔdd and LIΔdd:*dal* and found that the LIΔdd could not proliferate in phagocytic cells, and the proliferative ability of LIΔdd:*dal* was restored in phagocytic cells, but not fully recovered compared to LIΔ. On the one hand, the *dal* and *dat* genes may be involved in the regulation of virulence genes expression associated with *Listeria* escape from the phagosome of the host cells, and besides, this may also be relevant to the lower growth rate of LIΔdd:*dal* compared to LIΔ in vitro (Figure 2B). The leucine repeat residues (LRRs) in the LM InlA and InlB protein domains specifically bind to E‐cadherin and the hepatocyte growth factor receptor Met in non‐phagocytic cells to achieve internalization (Khelef et al., 2006; Pizarro‐Cerdá & Cossart, 2018). InlA is not found in LI, but the InlB protein family encoded by the LI *inlB‐1* and *inlB‐2* genes has a function similar to that of InlB (Domínguez‐Bernal et al., 2006). The ability of LIΔdd to invade hepatic cancer cells was significantly weakened, and the bacterial invasion ability recovered after D‐alanine supplementation or *dal* gene complementation compared to LIΔ (Figure 3D). Invasion and proliferative abilities are key for bacteria to be used as vaccine vectors. Therefore, the LI balanced‐lethal system holds promise for use as an antigen delivery vector.

LIΔdd:*dal* can colonize the liver and spleen of mice, but cannot sustain infection. The bacteria were cleared from the spleens after 2 days and from the liver after 5 days. This is may be attributed to the deletion of the *actA* and *plcB* genes, which may impair the ability of the bacteria to invade, replicate intracellularly and spread intra‐ and intercellularly. Specifically, *actA* and *plcB* are known to help *Listeria* escape phagosomes and infect adjacent cells (Engelbrecht et al., 1998; Pizarro‐Cerdá & Cossart, 2018). Our previous research showed that although LMΔ and LIΔ were significantly less virulent than the wild strains, the tuberculosis or foot‐and‐mouth disease viruses (FMDV) vaccines that were constructed using these attenuated strains retained immunogenicity (Lin et al., 2015; Mahdy, Liu, et al., 2019a; Mahdy, Sijing, et al., 2019b). Other studies reported that the LD50 of LMdd (knock out *dal* and *dat* genes based on wild‐type LM) was at least 10, 000‐fold lower than that of wild‐type LM (Thompson et al., 1998). Therefore, we speculated that the LD50 of the nutrient‐deficient strains constructed in this study may exceed 10^11^ CFU (the LD50 of LMΔ and LIΔ were approximately 10^7^ and 10^8^, respectively). Due to the limitation of dose for tail vein bacteria inoculation, we only measured the maximum safe dose of the nutrient‐deficient strains (5 × 10^8^ CFU). After complementation with *dal*, the virulence of LMΔdd:*dal* was slightly restored, and the mortality rate of the highest dose group (5 × 10^8^ CFU) was 0.2. Following 10 days of monitoring, we observed that mice from the LIΔdd:*dal* group survived the highest dose (5 × 10^8^ CFU/per mouse) of inoculation. Taken together, the nutrient‐deficient and complementation strains are largely attenuated, showing sufficient safety for their use as vaccine vectors.

We confirmed that combination therapy with two different recombinant *Listeria* strains (LM and LI) has a more satisfactory anti‐tumour effect than the administration of a single strain (Su et al., 2021). This suggests that both LI and LM hold strong potential for the development of *Listeria*‐vectored vaccines. Studies have shown that LMdd is immunogenic and can induce cellular immune responses (Sciaranghella et al., 2011; Whitney et al., 2011); however, there are no reports thus far regarding LI balanced‐lethal systems. In this study, we evaluated the immunogenicity and protective immune response induced by LIΔdd:*dal* in mice. Up to date, there are no reports on the T‐cell epitopes derived from LI; therefore, we expressed the ILO protein to evaluate the immune effect of LIΔ and LIΔdd:*dal*. Mice immunized with LIΔ or LIΔdd:*dal* showed specific T‐cell immune responses and the cytokine levels of T cells were significantly increased, especially in LIΔdd:*dal*‐immunized mice. Remarkably, LIΔdd:*dal*‐immunized mice showed a higher frequency of IL‐2 secreting T cells compared to PBS‐ or LIΔ‐immunized mice, particularly that of CD4^+^ cytokine‐secreting T cells. The cytokine IL‐2 is central to protective immunity and has shown a potent capacity to induce T‐cell growth in vitro and is an FDA‐approved drug for multiple metastatic cancers (Smith, 1988; Hsu et al., 2021). We challenged immunized mice with LIΔ and observed that the survival rate of mice immunized with LIΔdd:*dal* (25%) was higher than that of mice immunized with LIΔ (20%). In future applications, to maximize the protective efficacy of LIΔdd:*dal* vectored vaccines, the immunization strategy may need to be optimized and can be optimized by double boost immunization (Pownall et al., 2021) or co‐immunization with other type of vaccine. Besides, our previous research had confirmed that the sequential immunization with different *Listeria* strains vectored vaccine candidates will significantly enhance the immunotherapeutic effect (Su et al., 2021). So the protective efficacy could also be enhanced by co‐immunization with other vectored vaccine such as LMΔdd:*dal* vectored vaccine.

Overall, the balanced‐lethal systems (LIΔdd:*dal* and LMΔdd:*dal*) constructed in this study were genetically stable and their biochemical characteristics were similar to the parent strain. The LI balanced‐lethal system could proliferate in phagocytic cells and invade non‐phagocytic cells. Additionally, LIΔdd:*dal* was considerably attenuated, had enhanced immunogenicity in comparison with the parental strain and provided immune protection. This is the first report of an LI balanced‐lethal system, which advocates the use of such LI balanced‐lethal systems for vaccine development. In addition, we constructed an LM balanced‐lethal system using a homologous recombination protocol. We confirmed that the LM balanced‐lethal system can be stably inherited over multiple generations and that it is notably attenuated. This study has implications for live bacterial vaccine vector research on strain attenuation and heterologous antigen expression and provides vector candidates for sequential immunization.

## AUTHOR CONTRIBUTION

All authors contributed to the study conception and design. WC, LY and ZYZ conceived the study and designed the experiments. LY, ZYZ, LSJ and ZYW performed the experiments. LY, TSC, LT, HH and TT analysed the data and wrote the manuscript. All authors reviewed and approved the final version of the manuscript.

## FUNDING INFORMATION

National Natural Science Foundation of China under Scientific Research Project (No. 31570924).

## CONFLICT OF INTEREST

The authors declare that they have no conflict of interest.

## ETHICS STATEMENT

Mouse experiments were performed according to the guidelines of the Animal Care and Use Committee of Sichuan University (NO. Gwll2022073).

## Supporting information


Appendix S1

## Data Availability

The datasets generated and analysed during the current study are available from the corresponding author on reasonable request.
