# Supplementary material for: A Listeria ivanovii balanced‐lethal system may be a promising antigen carrier for vaccine construction
Source: Microb Biotechnol. 2022 Sep 7;15(11):2831–44. doi: 10.1111/1751-7915.14137 (PMC9618314; doi:10.1111/1751-7915.14137)
Supplement: Supplementary file 1 — Appendix S1 [file MBT2-15-2831-s001.pdf]

Microbial biotechnology

A *Listeria ivanovii* balanced-lethal system that might be a promising antigen carrier for vaccine construction

Yao Lei [1, 2#], Yuzhen Zhou[1, 2#], Yunwen Zhang[1, 2#], Sijing Liu [1, 2], Sicheng Tian[1, 2], Qian Ou[1, 2], Ting Liu[1, 2], Huan Huang[1, 2], Tian Tang[1, 2] and Chuan Wang[1, 2]*

Author details

[1]West China School of Public Health and West China Fourth Hospital, Sichuan University, Chengdu, P. R. China, Chengdu 610041, China;
[2]Research Center for Public Health and Preventive Medicine, West China School of Public Health, Sichuan University, Chengdu 610041, China.

Corresponding author

E-mail: wangchuan@scu.edu.cn
Phone No.: +86 18628297626
Fax: 86-28-85501272

Table. S1 The strains and plasmids used in this study

Strains or plasmids	Description	Reference of source
Bacteria		
LM	*Listeria monocytogenes* 10403S	Dr. Hao Shen; BEI Resources
LI	*Listeria ivanovii* PAM55	Dr. Hao Shen; ATCC
LMΔ*actAplcB* (LMΔ)	Knockdown of *atcA* and *plcB* genes in *Listeria monocytogenes* 10403S	Our lab
LIΔ*actAplcB* (LIΔ)	Knockdown of *atcA* and *plcB* genes in *Listeria ivanovii* PAM55	Our lab
Escherichia coli DH5α	General cloning strain	Takara
LMΔ*actAplcB*Δ*dal*Δ*dat* (LMΔdd)	Knockdown of *dal* and *dat* genes in LMΔ*atcAplcB*	This study
LIΔ*actAplcB*Δ*dal*Δ*dat* (LIΔdd)	Knockdown of *dal* and *dat* genes in LIΔ*atcAplcB*	This study
LMΔ*actAplcB*Δ*dal*Δ*dat*:*dal* (LMΔdd:*dal*)	LMΔdd harbouring antibiotic resistance-free plasmid pCW-GFP-LM*dal*	This study
LIΔ*atcAplcB*Δ*dal*Δ*dat*:*dal* (LIΔdd:*dal*)	LIΔdd harbouring antibiotic resistance-free plasmid pCW-GFP-LM*dal*	This study
Plasmids		

pCW619	Containing *Xba*I and *Spe*I single sites, Amp^R and Ery^R fragment	Our lab
pCW619-LM *dal*	pCW619 derivatives, possessed upstream and downstream homologous arms of LM *dal* gene	This study
pCW619-LI *dal*	pCW619 derivatives, possessed upstream and downstream homologous arms of LI *dal* gene	This study
pCW619-LM *dat*	pCW619 derivatives, possessed upstream and downstream homologous arms of LM *dat* gene	This study
pCW619-LI *dat*	pCW619 derivatives, possessed upstream and downstream homologous arms of LI *dat* gene	This study
pCW-GFP	Containing *Sap*I, *Ata*II, *Spe*I and *Sal*I single sites, Amp^R and Ery^R fragment, carrying gfp gene	(Zhang et al. 2019)
pCW-GFP-LM *dal*	pCW-GFP derivatives, with LM *dal* gene replacing the Ery^R fragment, and deleted with Amp^R	This study

Table. S2 Inoculation dose and mouse mortality

Strains	The Inoculation dose of *Listeria* (CFU/mouse)		
	1.8×10^8	3×10^8	5×10^8
LMΔdd	0	0	0
LIΔdd	0	0	0
LMΔdd:*dal*	0	0	0.2
LIΔdd:*dal*	0	0	0

Fig. S1

(A)

```
HomoLIdal-F/R.1 : TATTTGGTATTCTTCTACGTCGGACTTATTTACTATCCTGCGTTCATCATTCTATGTTATATTTTTAGGTTGTTTAGAATTCTACTACTCCTAGACTGAC :  100
HomoLIdal-F/R.0 : TATTTGGTATTCTTCTACGTCGGACTTATTTACTATCCTGCGTTCATCATTCTATGTTATATTTTTAGGTTGTTTAGAATTCTACTACTCCTAGACTGAC :  100

HomoLIdal-F/R.1 : TTCCAGCGCCTGGTTTACCTTGACCATTAGTTCCTCGTCCAAATGTGTAATCTTATCTGTCAGGCGTTGTTTGTCAATTGTTCTAATTTGTTCTAAAAGA :  200
HomoLIdal-F/R.0 : TTCCAGCGCCTGGTTTACCTTGACCATTAGTTCCTCGTCCAAATGTGTAATCTTATCTGTCAGGCGTTGTTTGTCAATTGTTCTAATTTGTTCTAAAAGA :  200

HomoLIdal-F/R.1 : ATGACAGAATCTCTTTCAAAGCCATCTTTACGAGTAGCTTCCACATGTGTTGGCAGCTTTGCTTTTTGAATCTTTGCGGTTATTGCTGCCACAATCACAG :  300
HomoLIdal-F/R.0 : ATGACAGAATCTCTTTCAAAGCCATCTTTACGAGTAGCTTCCACATGTGTTGGCAGCTTTGCTTTTTGAATCTTTGCGGTTATTGCTGCCACAATCACAG :  300

HomoLIdal-F/R.1 : TTGGGCTGAATCTATTACCAATGTCGTTTTGAATGATGAGAACAGGCCGTATTCCCCCTTGCTCGCTTCCGACCACGGGGGAAAGGTCCGCGTAGTATAC :  400
HomoLIdal-F/R.0 : TTGGGCTGAATCTATTACCAATGTCGTTTTGAATGATGAGAACAGGCCGTATTCCCCCTTGCTCGCTTCCGACCACGGGGGAAAGGTCCGCGTAGTATAC :  400

HomoLIdal-F/R.1 : GTCACCACGCTTCACCATCAGCCATTAACCTCCTAAAATACTAATATTTCTGTCTTCTGCTTCTGATTCGACATGGGTACACTCGCATGCGATAGCAAAG :  500
HomoLIdal-F/R.0 : GTCACCACGCTTCACCATCAGCCATTAACCTCCTAAAATACTAATATTTCTGTCTTCTGCTTCTGATTCGACATGGGTACACTCGCATGCGATAGCAAAG :  500

HomoLIdal-F/R.1 : TTAATTGTCGCCATTTCCGCATAGCCGCGCTCCATCTCGTCTCTCAACTCACGTGCCCTTTTCTCTTGCAAAAATTGTTGCGTTGCTTCCATTATAACTT :  600
HomoLIdal-F/R.0 : TTAATTGTCGCCATTTCCGCATAGCCGCGCTCCATCTCGTCTCTCAACTCACGTGCCCTTTTCTCTTGCAAAAATTGTTGCGTTGCTTCCATTATAACTT :  600

HomoLIdal-F/R.1 : CACTCCGCCCCATTTTTTCTTTTTCTACAACTACGTCGAGTTCCTGTACCATTTCTTGTGTCAGTTCTACGGATATTATCATCCGCTTTTCTTTCTCTAA :  700
HomoLIdal-F/R.0 : CACTCCGCCCCATTTTTTCTTTTTCTACAACTACGTCGAGTTCCTGTACCATTTCTTGTGTCAGTTCTACGGATATTATCATCCGCTTTTCTTTCTCTAA :  700

HomoLIdal-F/R.1 : CACGTGTCACACCCCCAAAGCCTCGTACCTGTAAGTAACAACTATATCGTACCATTATCAAGCCATGAAGAAAAGAGTTATTTAGCATTTACTTGTTAAG :  800
HomoLIdal-F/R.0 : CACGTGTCACACCCCCAAAGCCTCGTACCTGTAAGTAACAACTATATCGTACCATTATCAAGCCATGAAGAAAAGAGTTATTTAGCATTTACTTGTTAAG :  800

HomoLIdal-F/R.1 : ATTGTTATAGTTTTATCCAACATCCTCCCTTAAGTCCTAGATGTAGCTATAAAGTTTCATATGTTAGATTATAAACGCTTCTCCTCAAAAAAGCACGAAT :  900
HomoLIdal-F/R.0 : ATTGTTATAGTTTTATCCAACATCCTCCCTTAAGTCCTAGATGTAGCTATAAAGTTTCATATGTTAGATTATAAACGCTTCTCCTCAAAAAAGCACGAAT :  900

HomoLIdal-F/R.1 : ATTACCTT-------------------------------------------------------------------------------------------- :  908
HomoLIdal-F/R.0 : ATTACCTTCTAATGGATGTATTTTCTAGGTATACGCTCAGTTAGCATACAGGTGACTTCATAATTAATTGTGTCTAAATATTCAGCTGCATCATCAACTG : 1000

HomoLIdal-F/R.1 : ---------------------------------------------------------------------------------------------------- :    -
HomoLIdal-F/R.0 : TTACCGTGTTCTCTTGATCGGTACCAATTATCGTGACTTTTGAACCAGTTTGAAATTCATGTGGTAGTTTGATGATTGTTTGGTCCATACATACCCGCCC : 1100

HomoLIdal-F/R.1 : ---------------------------------------------------------------------------------------------------- :    -
HomoLIdal-F/R.0 : AACAATCGGAACTTTTTCTCCATTTACTAGTACGTGAAATCCACTGTAGTGACGAATAAGCCCATCTGCATAACCAATCGGCAATGTTGCTACCCATTCT : 1200

HomoLIdal-F/R.1 : ---------------------------------------------------------------------------------------------------- :    -
HomoLIdal-F/R.0 : TTTTCCGTTGCTGTATAGGTCGCTCCGTAGCTGACACTATCACCAGGCGCAAGTTCTTTCACATGAACCATCTCGGTATAAAGTGCAAGTGCCGGCTTTA : 1300

HomoLIdal-F/R.1 : ---------------------------------------------------------------------------------------------------- :    -
HomoLIdal-F/R.0 : GCTTAAATGGTAAACTTGCTTTAATTTCGATTGATGGCGTTAAACCATACATAGAAATTCCAAAACGAATCGCATCAAAGTCTACTTGAGATTGCAGTAA : 1400

HomoLIdal-F/R.1 : ---------------------------------------------------------------------------------------------------- :    -
HomoLIdal-F/R.0 : AGAGGCTGCTGAATTTGCCGTGTGGACAAATGTTGGTCGTTCGTTTAAAGCCCTTAAAATAGTATGGAATCTCACTAATTGTTGGTCAAAATAACTGGTT : 1500

HomoLIdal-F/R.1 : ---------------------------------------------------------------------------------------------------- :    -
HomoLIdal-F/R.0 : TCTAACTGATCAGCGGTAGCAAAATGCGTGTAGACTCCTTCTAAAATCAAATTCTTATTTTCAGCAATTTTCGCTTCGATTTGCGCCGCTTCTTGCGCCC : 1600
```

```
HomoLIdal-F/R.1 : ---------------------------------------------------------------------------------------------------- :    -
HomoLIdal-F/R.0 : CACGGATACCTAGACGCCCCATACCACTATCGATTTTCAAATGCACTTTTAATGGTACTTCAGGATTTACACCTTCTAACCAATCTTCTCTGTAAATCGT : 1700

HomoLIdal-F/R.1 : ---------------------------------------------------------------------------------------------------- :    -
HomoLIdal-F/R.0 : TAATGAAATATTTTTGCGTGCTGCTAGATTTGCATCTTCTTTTCTTGTTGCTCCTAAAACTAAAATAAAATCATCGCGAAAACCAGCTTCTCGTAAAGCC : 1800

HomoLIdal-F/R.1 : ---------------------------------------------------------------------------------------------------- :    -
HomoLIdal-F/R.0 : AGTGCTTCATCCAAAATAGCTACACAAAAGCCTTTTACTCCTGCTTCTTTGGCAGTTTTTGCCACTTCAATAATTCCATGACCATAGGCGTTTGCTTTCA : 1900

HomoLIdal-F/R.1 : ---------------------------------------------------------------------------------------------------- :    -
HomoLIdal-F/R.0 : CCACTGCCCAGACTGCTACGTTTTTTGGAAGACGTTTTTGTTCATTTTGGATGTTTTCACGTATTGCTGCACAGTCGATTTCAATCCATGTTGGACGATG : 2000

HomoLIdal-F/R.1 : ---------------AATTTCCACATTCCTTTCTAAACTTCAATAATCACTTGTGCCGCAGCAGATCTCGCCGTGTGGGTAATACTCACAAACACCGACT :  993
HomoLIdal-F/R.0 : CCAGCCTGTCACCATAATTTCCACATTCCTTTCTAAACTTCAATAATCACTTGTGCCGCAGCAGATCTCGCCGTGTGGGTAATACTCACAAACACCGACT : 2100

HomoLIdal-F/R.1 : CACCAGGTTTGATCGGCATTGTCACGTGAGGTCTGCCATCTTCTACTTGTAAAATTTCTACATCCGTAAAGCTTAAATGTTTTCCAAATCCAGTTCCGTT : 1093
HomoLIdal-F/R.0 : CACCAGGTTTGATCGGCATTGTCACGTGAGGTCTGCCATCTTCTACTTGTAAAATTTCTACATCCGTAAAGCTTAAATGTTTTCCAAATCCAGTTCCGTT : 2200

HomoLIdal-F/R.1 : CGCTTTTGCATAAGCTTCTTTGGCAGCAAAACGCCCCGCTAAAAAATTCAATTTTACGACTACCTTCATATTTTTCAAATTGGCTAATTTCTTTTTCTGTT : 1193
HomoLIdal-F/R.0 : CGCTTTTGCATAAGCTTCTTTGGCAGCAAAACGCCCCGCTAAAAAATTCAATTTTACGACTACCTTCATATTTTTCAAATTGGCTAATTTCTTTTTCTGTT : 2300

HomoLIdal-F/R.1 : AAAATCCGCTCCATAAAACGGGGATTCTTTTCTAAAACTTGTTTTACTCGTGCTAAATCAATCATATCAAGCCCAATACCTTTAATCATCTTACACCTCT : 1293
HomoLIdal-F/R.0 : AAAATCCGCTCCATAAAACGGGGATTCTTTTCTAAAACTTGTTTTACTCGTGCTAAATCAATCATATCAAGCCCAATACCTTTAATCATCTTACACCTCT : 2400

HomoLIdal-F/R.1 : TTCAAAAAAATCAGTAACGTCATTAACAGCTAGTTTCGCCCCAGCAATACAATCAGGAATTCCAACACCTTGATAACTCATTCCTGCAAAATAAATTCCCG : 1393
HomoLIdal-F/R.0 : TTCAAAAAAATCAGTAACGTCATTAACAGCTAGTTTCGCCCCAGCAATACAATCAGGAATTCCAACACCTTGATAACTCATTCCTGCAAAATAAATTCCCG : 2500

HomoLIdal-F/R.1 : GGTAGTCTGTCGCAATATTTTCTTTTAGTTGTTTTAGTCTAGCTTGGTGTGTCACTAAATACTGTGGCATTGCAGACTTCATCCGACTTACTTCATAAAA : 1493
HomoLIdal-F/R.0 : GGTAGTCTGTCGCAATATTTTCTTTTAGTTGTTTTAGTCTAGCTTGGTGTGTCACTAAATACTGTGGCATTGCAGACTTCATCCGACTTACTTCATAAAA : 2600

HomoLIdal-F/R.1 : AAGTGGCGCTCTTTCGATATCCATCATTTCGGCATAATCTGCTAAAACAGCAGAAATAATTGCTTCATCACTTGCCTCTTCTAACCAAGTTTCACCTGCT : 1593
HomoLIdal-F/R.0 : AAGTGGCGCTCTTTCGATATCCATCATTTCGGCATAATCTGCTAAAACAGCAGAAATAATTGCTTCATCACTTGCCTCTTCTAACCAAGTTTCACCTGCT : 2700

HomoLIdal-F/R.1 : TTACCAACAAAACCTCGTAGTAGCATTTTATTTTCAGGTACCATATGTGGCCATTTTTTTTGCACCCATGTACAAGCAGTGGTTCGATAAGGAGCAGTTC : 1693
HomoLIdal-F/R.0 : TTACCAACAAAACCTCGTAGTAGCATTTTATTTTCAGGTACCATATGTGGCCATTTTTTTTGCACCCATGTACAAGCAGTGGTTCGATAAGGAGCAGTTC : 2800

HomoLIdal-F/R.1 : TAGCAACTAAATAACCCGTTCCATCCGGTAAAATGGGTACATCACTTTCATTATAAGCTAATGAGACTGTTGCAAGGCTGGTTAATGGCTGGTTGGCAAA : 1793
HomoLIdal-F/R.0 : TAGCAACTAAATAACCCGTTCCATCCGGTAAAATGGGTACATCACTTTCATTATAAGCTAATGAGACTGTTGCAAGGCTGGTTAATGGCTGGTTGGCAAA : 2900

HomoLIdal-F/R.1 : CGGTTCTGTTACCCTGGCTCCAAGTAAATCAATTAATGTATCGTGAGTAGCAGCAATAATGACGCCATCAGCAGATATTTTATCTCCATTTTCAAAAAAA : 1893
HomoLIdal-F/R.0 : CGGTTCTGTTACCCTGGCTCCAAGTAAATCAATTAATGTATCGTGAGTAGCAGCAATAATGACGCCATCAGCAGATATTTTATCTCCATTTTCAAAAAAA : 3000

HomoLIdal-F/R.1 : ATTTCATAAGAATGACCTTTTTTCAAAATTTGCGTTGCTTGTTTTC------------------------------------------------------ : 1939
HomoLIdal-F/R.0 : ATTTCATAAGAATGACCTTTTTTCAAAATTTGCGTTGCTTGTTTTC------------------------------------------------------ : 3046
```

(B)

```
HomoLIdat-F/R.1 : ATGCATTTCTTTTGATGATTTTCCTGCATTTTTGATGGCAAGTTTCCCACCATCGAGTAGGTCGTCTCCGACTCGCAACATTTTGGAACCTGCGCTCGCG :  100
HomoLIdat-F/R.0 : ATGCATTTCTTTTGATGATTTTCCTGCATTTTTGATGGCAAGTTTCCCACCATCGAGTAGGTCGTCTCCGACTCGCAACATTTTGGAACCTGCGCTCGCG :  100

HomoLIdat-F/R.1 : ATTTTGGTGCCGGAACTAAAGGCTTTGACTCCTGGTATAATATCGAGTAAGGCAAATCCTCCTCGGAGCACGCGTTCCTGGTCGCTTAGTTCTCGGCCGG :  200
HomoLIdat-F/R.0 : ATTTTGGTGCCGGAACTAAAGGCTTTGACTCCTGGTATAATATCGAGTAAGGCAAATCCTCCTCGGAGCACGCGTTCCTGGTCGCTTAGTTCTCGGCCGG :  200

HomoLIdat-F/R.1 : ATATCCAATCTTTTCCAGTAACTGCTGAAGAGGCTTCTAGTGCGCCAAATCCTACTCCAAGTGCTAATCCGGCTGGTGGGCAGACGATACTTACGCCCAC :  300
HomoLIdat-F/R.0 : ATATCCAATCTTTTCCAGTAACTGCTGAAGAGGCTTCTAGTGCGCCAAATCCTACTCCAAGTGCTAATCCGGCTGGTGGGCAGACGATACTTACGCCCAC :  300

HomoLIdat-F/R.1 : AATCACCACGGCGGCAACGATATTGACCCAGAACTCTTTCTCTTCTTGTTCGTCTTGGACGGATTTATAATCAAACGCACGAGTATGTAACATCCCCATT :  400
HomoLIdat-F/R.0 : AATCACCACGGCGGCAACGATATTGACCCAGAACTCTTTCTCTTCTTGTTCGTCTTGGACGGATTTATAATCAAACGCACGAGTATGTAACATCCCCATT :  400

HomoLIdat-F/R.1 : TTAAAATCGGCTTCGCTAACCTTTTGGTCCGGATTTTCATGCTTCCAGACTTCGTATTGGAGGGTCATTTGGTTGGCGTAGAAGTTATCGCCATTCATTA :  500
HomoLIdat-F/R.0 : TTAAAATCGGCTTCGCTAACCTTTTGGTCCGGATTTTCATGCTTCCAGACTTCGTATTGGAGGGTCATTTGGTTGGCGTAGAAGTTATCGCCATTCATTA :  500

HomoLIdat-F/R.1 : AGTCCTCAATAGTTGCATTTTCTTTTACCGATTCAATCACTTGGGGCTGATTACTGTACGAAGACATATACGTTTGTGTCGACTTAGCCCCAATACGGTT :  600
HomoLIdat-F/R.0 : AGTCCTCAATAGTTGCATTTTCTTTTACCGATTCAATCACTTGGGGCTGATTACTGTACGAAGACATATACGTTTGTGTCGACTTAGCCCCAATACGGTT :  600

HomoLIdat-F/R.1 : TGTAGTTGAGTATGTCGTGATACTCAAGTCTTGCATCCCATCTACAAATGCATCCAACTTCTTGTAAAAAGGCTCATCAATCAGCGTTTCGATTTTCTCC :  700
HomoLIdat-F/R.0 : TGTAGTTGAGTATGTCGTGATACTCAAGTCTTGCATCCCATCTACAAATGCATCCAACTTCTTGTAAAAAGGCTCATCAATCAGCGTTTCGATTTTCTCC :  700

HomoLIdat-F/R.1 : CCTGCGTGACAACTAAACTGATACAGCACCTTTAAGTCTTCCTCGATTTCCCCGTACTTGCTTCGATGGTCGGTATGTTGGAAATGAATCGCGTGATCGG :  800
HomoLIdat-F/R.0 : CCTGCGTGACAACTAAACTGATACAGCACCTTTAAGTCTTCCTCGATTTCCCCGTACTTGCTTCGATGGTCGGTATGTTGGAAATGAATCGCGTGATCGG :  800

HomoLIdat-F/R.1 : TGTCTAAGTCACGGATATCGTCTTCCGCATTCTCCATATTTTTCGACACTTTTTCTAAATGATTAAATAGGCCACCAAACCAATTGGCTGCATTAAATGG :  900
HomoLIdat-F/R.0 : TGTCTAAGTCACGGATATCGTCTTCCGCATTCTCCATATTTTTCGACACTTTTTCTAAATGATTAAATAGGCCACCAAACCAATTGGCTGCATTAAATGG :  900

HomoLIdat-F/R.1 : AGCTAGGGCACTTTTGGCTTTTTACCAGTCGTCTTCTCGGTAGTTTACGTCTTTCATTCATCTGTCCTCGACTTTCTTTGTTATATGAGCTGTATATTAA : 1000
HomoLIdat-F/R.0 : AGCTAGGGCACTTTTGGCTTTTTACCAGTCGTCTTCTCGGTAGTTTACGTCTTTCATTCATCTGTCCTCGACTTTCTTTGTTATATGAGCTGTATATTAA : 1000

HomoLIdat-F/R.1 : TGAAAGTTCATCTCCAAATCTTGTTATATGTTTCTTTTAATCAATATAAGCAACCATCCTTTTAAGATGGTTGCCCAAAC-------------------- : 1080
HomoLIdat-F/R.0 : TGAAAGTTCATCTCCAAATCTTGTTATATGTTTCTTTTAATCAATATAAGCAACCATCCTTTTAAGATGGTTGCCCAAACTATAAAAAACAGAGATTCTC : 1100

HomoLIdat-F/R.1 : ---------------------------------------------------------------------------------------------------- :    -
HomoLIdat-F/R.0 : GTTATTTAGTATTCGAGGATTTTTGTTTTTCTACAGTCTGAAAGCCTTCCTCAAAAAAGGAAGACTTTGACTTTAAATTATTTAGCTAATACTAATTCAC : 1200

HomoLIdat-F/R.1 : ---------------------------------------------------------------------------------------------------- :    -
HomoLIdat-F/R.0 : CACAAGAACGAACAATTTCTTCCACAAAATAGCCATGAAGTTGCGCGGTAATTGGACCACGTTTTCCGTCACCTACTTGTACGCCATCAATATGTGTAAT : 1300

HomoLIdat-F/R.1 : ---------------------------------------------------------------------------------------------------- :    -
HomoLIdat-F/R.0 : TGGCGTAATTTCGATTGTTGTGCTAGAAATAAACACTTCATCTGCTTCTCGAAGGTCAGTTAGTGTAAAATCTGCTTCTCGGACTGGAATACCATTTTTT : 1400

HomoLIdat-F/R.1 : ---------------------------------------------------------------------------------------------------- :    -
HomoLIdat-F/R.0 : CTAGCAACATCTAAAATAACTTGACGAGTAATTCCGTTTAAAATTAAATTATCAGCTGCATGTGTCCAAAGCACACCATCTTTAATAATAGAAACATTGG : 1500

HomoLIdat-F/R.1 : ---------------------------------------------------------------------------------------------------- :    -
HomoLIdat-F/R.0 : ATGCGGAACATTCGGTAACTTGTTCACCGCGGTGCAAAATAGCTTCTAAAGCATCTTGTTGATGTGCTTTATTCTTGGCCATAATGTTTCCAAGTAAGCT : 1600

HomoLIdat-F/R.1 : ---------------------------------------------------------------------------------------------------- :    -
HomoLIdat-F/R.0 : TAAACTTTTAATGTCACAACGTAACCAGCGCACATCTTCTTCTGTAATCGCTGTTCCACCTTCGATAAATTGTTTTTCATTTCTAGGAACCTCGCGAGCT : 1700
```

```
HomoLIdat-F/R.1 : ---------------------------------------------------------------------------------------------------- :    -
HomoLIdat-F/R.0 : GCTGCTGTTAGTACTCCTTCTAATGGGAAATCATCTGGAATCACATGGTTACGAGGATTTTGGACGCCACGAGTTACCTGTAAATAAACATTTCCTGTAT : 1800

HomoLIdat-F/R.1 : ---------------------------------------------------------------------------------------------------- :    -
HomoLIdat-F/R.0 : GAATATTATTTGCCGCCACTAACTCTTCAATTAATTTACGTAGTTCTTCTTTGGAATATGGTATGACTAAATCAATTTTTGCAGCGCTGGCATATAAACG : 1900

HomoLIdat-F/R.1 : ---------------------------------------------------------------------------------------------------- :    -
HomoLIdat-F/R.0 : GTCGATATGTTCATCATATGTAAAGAATTTGCCATTATACAAACGAACAACTTCATATACCCCATCTCCAAATTGATATCCGCGGTCTTCCACGTCTACG : 2000

HomoLIdat-F/R.1 : -----------------------------------------------CTCAAGACACTCCTTGCTAGTTAATTTATTGGAACTATCTAAAAACTTAATTA : 1133
HomoLIdat-F/R.0 : GTCGCATCTTCTCTTTCCACTAAATGGTTATTTACTAATACTTTCATCTCAAGACACTCCTTGCTAGTTAATTTATTGGAACTATCTAAAAACTTAATTA : 2100

HomoLIdat-F/R.1 : AATAGAGAAACTGCCGCAAAACTAAGCTTTGCAGCAGTTAATTTTTTCAATTATAACCCTTACTTTGCTAATTTGTAAAGCGCTTCTGCGTAAATTGCTG : 1233
HomoLIdat-F/R.0 : AATAGAGAAACTGCCGCAAAACTAAGCTTTGCAGCAGTTAATTTTTTCAATTATAACCCTTACTTTGCTAATTTGTAAAGCGCTTCTGCGTAAATTGCTG : 2200

HomoLIdat-F/R.1 : TCGCTTTTAACAAATCGTCAAAATAACTGAATTCGTCTTTTTGGTGCATCGTATCTTCGCGGCCTGGGAATAGTGCGCCAAACGCCACACCTGTTTCCAT : 1333
HomoLIdat-F/R.0 : TCGCTTTTAACAAATCGTCAAAATAACTGAATTCGTCTTTTTGGTGCATCGTATCTTCGCGGCCTGGGAATAGTGCGCCAAACGCCACACCTGTTTCCAT : 2300

HomoLIdat-F/R.1 : ATGACGTGCATAAGTTCCGCCACCAATTGCAAGTAAAGTAGCTTCTTCTCCTGTTTGTTTCGTATAAACTTCTTGTAAAGTTTGAATTAATGGGTGGTCT : 1433
HomoLIdat-F/R.0 : ATGACGTGCATAAGTTCCGCCACCAATTGCAAGTAAAGTAGCTTCTTCTCCTGTTTGTTTCGTATAAACTTCTTGTAAAGTTTGAATTAATGGGTGGTCT : 2400

HomoLIdat-F/R.1 : TTTGGTACGAAAAGCGGTTTGGAATCACTGTAATGTGTGTACTGTGCATTATATTCATATACGACAGTTTGCATTTTATTTTTCAGCTTATCCATGTTTG : 1533
HomoLIdat-F/R.0 : TTTGGTACGAAAAGCGGTTTGGAATCACTGTAATGTGTGTACTGTGCATTATATTCATATACGACAGTTTGCATTTTATTTTTCAGCTTATCCATGTTTG : 2500

HomoLIdat-F/R.1 : CAGTAACTGGGTAACGGAAATTAAGTCCGTATTTCCCACCTTCCCCAACATCATAACGGATAACGCCAACATTCATCGTTAATTCGCCACTTTCTTTATC : 1633
HomoLIdat-F/R.0 : CAGTAACTGGGTAACGGAAATTAAGTCCGTATTTCCCACCTTCCCCAACATCATAACGGATAACGCCAACATTCATCGTTAATTCGCCACTTTCTTTATC : 2600

HomoLIdat-F/R.1 : TTCGTAGCTAATACCAAGTTTTACAGCACGAGAATCGCCAAATAAGTAATCACGACCAAATGTAACGAAATCATTAGCAGCACCAGTTAATTTAAATTTA : 1733
HomoLIdat-F/R.0 : TTCGTAGCTAATACCAAGTTTTACAGCACGAGAATCGCCAAATAAGTAATCACGACCAAATGTAACGAAATCATTAGCAGCACCAGTTAATTTAAATTTA : 2700

HomoLIdat-F/R.1 : CCTAAGAAGGCTACTAAATGAAGACCTGCATTGATACCATTATTTGGTTCCATTGCATGAGCAGATTTTCCAACCATGTTAATTTTAACTGTTTTACCGT : 1833
HomoLIdat-F/R.0 : CCTAAGAAGGCTACTAAATGAAGACCTGCATTGATACCATTATTTGGTTCCATTGCATGAGCAGATTTTCCAACCATGTTAATTTTAACTGTTTTACCGT : 2800

HomoLIdat-F/R.1 : CTTCTTCTAACGTACCTTCTACTGGATGGTTAGCTAAGAAAGTTTTGAAAGTACTTGTTAGTTTATCGTAGTCTTTCACGTTTTCGATGATGGCACTAGC : 1933
HomoLIdat-F/R.0 : CTTCTTCTAACGTACCTTCTACTGGATGGTTAGCTAAGAAAGTTTTGAAAGTACTTGTTAGTTTATCGTAGTCTTTCACGTTTTCGATGATGGCACTAGC : 2900

HomoLIdat-F/R.1 : ATGATCTGGTACCATATTGTAACGCTCACCTGACTCAAAGCTTAGTAAGCGGAATGCAGCTTCCCCGCTTGCTTCTCCGTCTTTAAAAGATACATCTAAT : 2033
HomoLIdat-F/R.0 : ATGATCTGGTACCATATTGTAACGCTCACCTGACTCAAAGCTTAGTAAGCGGAATGCAGCTTCCCCGCTTGCTTCTCCGTCTTTAAAAGATACATCTAAT : 3000

HomoLIdat-F/R.1 : TCAGAAATACCTTTTTCCGCGTGAATAATCGGGAATTCGGCATCAGGAACAAAACCGAGTGTTGGTTGTTCTTCTGTTTCGAAATAACGTTCCACACAGC : 2133
HomoLIdat-F/R.0 : TCAGAAATACCTTTTTCCGCGTGAATAATCGGGAATTCGGCATCAGGAACAAAACCGAGTGTTGGTTGTTCTTCTGTTTCGAAATAACGTTCCACACAGC : 3100

HomoLIdat-F/R.1 : TCATACCGCTTCTCATCAGACCCACCCATCA--------------------------------------------------------------------- : 2164
HomoLIdat-F/R.0 : TCATACCGCTTCTCATCAGACCCACCCATCA--------------------------------------------------------------------- : 3131
```

(C)

```
LMdal.1 : ATGGTGACAGGCTGGCATCGTCCAACATGGATTGAAATAGACCGCGCAGCAATTCGCGAAAATATAAAAAATGAACAAAATAAACTCCCGGAAAGTGTCG :  100
LMdal.0 : ATGGTGACAGGCTGGCATCGTCCAACATGGATTGAAATAGACCGCGCAGCAATTCGCGAAAATATAAAAAATGAACAAAATAAACTCCCGGAAAGTGTCG :  100
```

```
LMdal.1 : ACTTATGGGCAGTAGTCAAAGCTAATGCATATGGTCACGGAATTATCGAAGTTGCTAGGACGGCGAAAGAAGCTGGAGCAAAAGGTTTCTGCGTAGCCAT :  200
LMdal.0 : ACTTATGGGCAGTAGTCAAAGCTAATGCATATGGTCACGGAATTATCGAAGTTGCTAGGACGGCGAAAGAAGCTGGAGCAAAAGGTTTCTGCGTAGCCAT :  200

LMdal.1 : TTTAGATGAGGCACTGGCTCTTAGAGAAGCTGGATTTCAAGATGACTTTATTCTTGTGCTTGGTGCAACCAGAAAAGAAGATGCTAATCTGGCAGCCAAA :  300
LMdal.0 : TTTAGATGAGGCACTGGCTCTTAGAGAAGCTGGATTTCAAGATGACTTTATTCTTGTGCTTGGTGCAACCAGAAAAGAAGATGCTAATCTGGCAGCCAAA :  300

LMdal.1 : AACCACATTTCACTTACTGTTTTTAGAGAAGATTGGCTAGAGAATCTAACGCTAGAAGCAACACTTCGAATTCATTTAAAAGTAGATAGCGGTATGGGGC :  400
LMdal.0 : AACCACATTTCACTTACTGTTTTTAGAGAAGATTGGCTAGAGAATCTAACGCTAGAAGCAACACTTCGAATTCATTTAAAAGTAGATAGCGGTATGGGGC :  400

LMdal.1 : GTCTCGGTATTCGTACGACTGAAGAAGCACGGCGAATTGAAGCAACCAGTACTAATGATCACCAATTACAACTGGAAGGTATTTACACGCATTTTGCAAC :  500
LMdal.0 : GTCTCGGTATTCGTACGACTGAAGAAGCACGGCGAATTGAAGCAACCAGTACTAATGATCACCAATTACAACTGGAAGGTATTTACACGCATTTTGCAAC :  500

LMdal.1 : AGCCGACCAGCTAGAAACTAGTTATTTTGAACAACAATTAGCTAAGTTCCAAACGATTTTAACGAGTTTAAAAAAACGACCAACTTATGTTCATACAGCC :  600
LMdal.0 : AGCCGACCAGCTAGAAACTAGTTATTTTGAACAACAATTAGCTAAGTTCCAAACGATTTTAACGAGTTTAAAAAAACGACCAACTTATGTTCATACAGCC :  600

LMdal.1 : AATTCAGCTGCTTCATTGTTACAGCCACAAATCGGGTTTGATGCGATTCGCTTTGGTATTTCGATGTATGGATTAACTCCCTCCACAGAAATCAAAACTA :  700
LMdal.0 : AATTCAGCTGCTTCATTGTTACAGCCACAAATCGGGTTTGATGCGATTCGCTTTGGTATTTCGATGTATGGATTAACTCCCTCCACAGAAATCAAAACTA :  700

LMdal.1 : GCTTGCCGTTTGAGCTTAAACCTGCACTTGCACTCTATACCGAGATGGTTCATGTGAAAGAACTTGCACCAGGCGATAGTGTTAGCTACGGAGCAACTTA :  800
LMdal.0 : GCTTGCCGTTTGAGCTTAAACCTGCACTTGCACTCTATACCGAGATGGTTCATGTGAAAGAACTTGCACCAGGCGATAGTGTTAGCTACGGAGCAACTTA :  800

LMdal.1 : TACAGCAACAGAGCGAGAATGGGTTGCGACATTACCAATTGGCTATGCGGATGGATTGATTCGTCATTACAGTGGTTTCCATGTTTTAGTAGACGGTGAA :  900
LMdal.0 : TACAGCAACAGAGCGAGAATGGGTTGCGACATTACCAATTGGCTATGCGGATGGATTGATTCGTCATTACAGTGGTTTCCATGTTTTAGTAGACGGTGAA :  900

LMdal.1 : CCAGCTCCAATCATTGGTCGAGTTTGTATGGATCAAACCATCATAAAACTACCACGTGAATTTCAAACTGGTTCAAAAGTAACGATAATTGGCAAAGATC : 1000
LMdal.0 : CCAGCTCCAATCATTGGTCGAGTTTGTATGGATCAAACCATCATAAAACTACCACGTGAATTTCAAACTGGTTCAAAAGTAACGATAATTGGCAAAGATC : 1000

LMdal.1 : ATGGTAACACGGTAACAGCAGATGATGCCGCTCAATATTTAGATACAATTAATTATGAGGTAACTTGTTTGTTAAATGAGCGCATACCTAGAAAATACAT : 1100
LMdal.0 : ATGGTAACACGGTAACAGCAGATGATGCCGCTCAATATTTAGATACAATTAATTATGAGGTAACTTGTTTGTTAAATGAGCGCATACCTAGAAAATACAT : 1100

LMdal.1 : CCATTAG : 1107
LMdal.0 : CCATTAG : 1107
```

Fig. S1 Analysis of the sequencing results of LIΔdd:*dal* that recovered from mice liver and the complement plasmid. (A) The nucleotide sequences alignment between PCR product (HomoLIdal-F/R.1) and the sequence of standard strains (HomoLIdal-F/R.0). (B) The nucleotide sequences alignment between PCR product (HomoLIdat-F/R.1) and the sequence of standard strains (HomoLIdat-F/R.0). (C) The nucleotide sequences alignment between the *dal* gene sequence of plasmid (LMdal.1) and the expected sequence of PCW-GFP-LM *dal* (LMdal.0). Black area means no mutation in nucleotide sequences. Due to the instability of sequencing, it is common that there are differences in the first and last 20 nucleotides, the sequencing results presented here have removed about 20 nucleotides of the beginning and the end of the sequences, respectively. HomoLIdal-F/R.1 and HomoLIdat-F/R.1: Sequencing of PCR products from colonies on BHI plates of liver; LMdal.1: Sequencing of extracted complement plasmid; HomoLIdal-F/R.0 and HomoLIdat-F/R.0: Sequence of standard

strains (LI PAM55; NCBI accession no. NC_016011.1); LMdal.0: Sequence of pCW-GFP-LM *dal* plasmid (NCBI accession no. MN513050). The sequencing results of LIΔdd:*dal* recovered from mouse liver with D-BHI plates and from mouse spleen with BHI/D-BHI plates were completely consistent with Fig. S1.
